# WNT5a export onto extracellular vesicles studied at single‐molecule and single‐vesicle resolution

**DOI:** 10.1111/febs.70074

**Published:** 2025-04-01

**Authors:** Antonia Schubert, Ajaree Mongkolsittisilp, Andrei Kobitski, Matthias Schulz, Oksana Voloshanenko, Meike Schaffrinski, Nadine Winkler, Michelle Neßling, Karsten Richter, Dominique Kranz, Karin Nienhaus, Dirk Jäger, Lorenz Trümper, Judith Büntzel, Claudia Binder, Gerd Ulrich Nienhaus, Michael Boutros

**Affiliations:** ^1^ Division Signaling and Functional Genomics German Cancer Research Center (DKFZ) Heidelberg Germany; ^2^ Department of Cell and Molecular Biology, Medical Faculty Mannheim Heidelberg University Heidelberg Germany; ^3^ Institute for Human Genetics, Medical Faculty Heidelberg Heidelberg University Heidelberg Germany; ^4^ Department of Hematology and Medical Oncology University Medical Center Göttingen Germany; ^5^ Department of Medical Oncology, National Center for Tumor Diseases (NCT) University Hospital Heidelberg Germany; ^6^ Institute of Applied Physics Karlsruhe Institute of Technology Karlsruhe Germany; ^7^ Central Unit Electron Microscopy German Cancer Research Center (DKFZ) Heidelberg Germany; ^8^ Institute of Nanotechnology Karlsruhe Institute of Technology Karlsruhe Germany; ^9^ Institute of Biological and Chemical Systems Karlsruhe Institute of Technology Karlsruhe Germany; ^10^ Department of Physics University of Illinois at Urbana‐Champaign Urbana IL USA

**Keywords:** extracellular vesicles, fluorescence correlation spectroscopy, number and brightness analysis, WNT signaling, WNT transport

## Abstract

WNT signaling governs development, homeostasis, and aging of cells and tissues, and is frequently dysregulated in pathophysiological processes such as cancer. WNT proteins are hydrophobic and traverse the intercellular space between the secreting and receiving cells on various carriers, including extracellular vesicles (EVs). Here, we address the relevance of different EV fractions and other vehicles for WNT5a protein, a non‐canonical WNT ligand that signals independently of beta‐catenin. Its highly context‐dependent roles in cancer (either tumor‐suppressive or tumor‐promoting) have been attributed to two distinct isoforms, WNT5a Short (WNT5aS) and WNT5a Long (WNT5aL), resulting from different signal peptide cleavage sites. To explore possible differences in secretion and extracellular transport, we developed fusion constructs with the fluorescent proteins (FPs) mScarlet and mOxNeonGreen. Functional reporter assays revealed that both WNT5a isoforms inhibit canonical WNT signaling, and EVs produced by WNT5a‐bearing tumor cells, carrying either of the WNT5a isoforms, induced invasiveness of the luminal A breast cancer cell line MCF7. We used fluorescence intensity distribution analysis (FIDA) and fluorescence correlation spectroscopy (FCS) to characterize at single‐molecule sensitivity WNT5aL‐bearing entities secreted by HEK293T cells. Importantly, we found that most WNT5aL proteins remained monomeric in the supernatant after ultracentrifugation; only a minor fraction was EV‐bound. We further determined the average sizes of the EV fractions and the average number of WNT5aL proteins per EV. Our detailed biophysical analysis of the physical nature of the EV populations is an important step toward understanding context‐dependent WNT cargo loading and signaling in future studies.

## Graphical abstract

WNT signaling, which orchestrates tissue development and homeostasis, is mediated by hydrophobic WNT proteins traveling from secreting to receiving cells on various carriers. Here, fusion constructs of fluorescent proteins with WNT5a were developed and functionally validated. By using quantitative fluorescence microscopy at the single‐particle level, secreted WNT5a carriers were characterized, which is essential for assessing their relevance for WNT signaling.

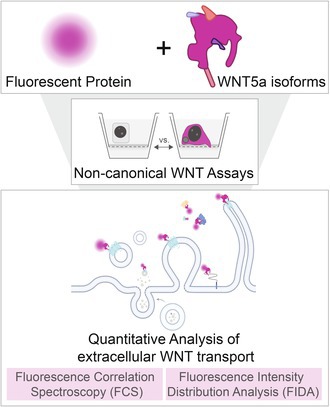

**Abbreviations**
Alix: apoptosis‐linked gene 2‐interacting protein X
Arf6: ADP‐ribosylation factor 6
CD9/63/81: cluster of differentiation 9/63/81 (Tetraspanins)
CM: conditioned medium
DLS: dynamic light scattering
Dvl: disheveled
(e)GFP: (Enhanced) Green Fluorescent Protein
ESCRT: endosomal sorting complex required for transport
Evi: WNT Cargo receptor evenness interrupted
EVs: extracellular vesicles
Exos: exosomes
FCS: fluorescence correlation spectroscopy
FIDA: fluorescence intensity distribution analysis
FPs: fluorescent proteins
Fzd: Frizzled (WNT receptor)
HEK293T: human embryonic kidney 293T cells
HSPGs: heparan sulfate proteoglycans
lEVs: large extracellular vesicles
MCF7: luminal A breast cancer cell line
mOxNeonGreen/mOxNG: monomeric OxNeonGreen fluorescent protein
mScarlet/mSc: monomeric scarlet fluorescent protein
MVBs: multivesicular bodies
MVs: microvesicles
nt: non‐tagged
NTA: nanoparticle tracking analysis
P100k: pellet obtained at 100 000 ***g***
P14k: pellet obtained at 14 000 ***g***
PCH: photon counting histogram
PCP: planar cell polarity
Rabs: Ras‐associated binding proteins
RFP: red fluorescent protein
ROR1/2: receptor tyrosine kinase‐like orphan receptor
RTK: receptor tyrosine kinase
rWNT: recombinant WNT
sEVs: small extracellular vesicles
sFRP2: secreted frizzled‐related protein 2
SN100k: supernatant obtained after centrifugation at 100 000 ***g***
SN14k: supernatant obtained after centrifugation at 14 000 ***g***
TCF4: transcription factor 4
TEM: transmission electron microscopy
WNT: wingless/integrated
WNT3(a): WNT3 (canonical WNT ligand)
WNT5a: WNT5a protein
WNT5aL: WNT5a long
WNT5aS: WNT5a short
YKT6: synaptobrevin homolog YKT6

## Introduction

WNT signaling drives tissue development and regeneration and is frequently dysregulated in cancer. In mammals, 19 WNT proteins act as extracellular ligands in WNT signaling. They all share 22 to 24 conserved cysteine residues [1] and are post‐translationally modified by palmitoylation and glycosylation [2, 3, 4]. As like most secreted proteins, WNT precursors have an N‐terminal signal peptide that is cleaved off during the secretory cascade to produce the mature polypeptide [5, 6, 7].

WNTs can activate signaling in cells distant from their source of secretion [8]. Due to their hydrophobic properties, WNT ligands need lipid‐shielding carriers in the extracellular aqueous milieu. Proposed mechanisms for WNT transport in the extracellular space, varying in their range of signaling, include WNT‐binding chaperones, heparan sulfate proteoglycans (HSPGs), lipoproteins, and transport via cytonemes (as reviewed in [9]). Furthermore, it has recently been shown that WNT ligands can also be transported on at least two types of extracellular vesicles (EVs): small and large EVs [10, 11, 12, 13, 14, 15]. However, the biological role of EVs in WNT signaling is still under debate (as reviewed in [16]).

Larger EVs (lEVs, 100–1000 nm diameter) usually directly bud from the outer plasma membrane and were commonly denoted microvesicles (MVs), ectosomes, or microparticles [17]. Cargo and composition of lEVs reflect those of the cellular cytoplasm and the outer plasma membrane. Small EVs (sEVs, 30–100 nm in diameter) usually derive from the endosomal compartment and were also classified as exosomes (Exos) [17]. They are formed as intraluminal vesicles in multivesicular bodies (MVBs) through an invagination of the MVB membrane after ESCRT (endosomal sorting complex required for transport) dependent or independent cargo clustering [17]. sEVs are released into the extracellular environment by fusing the MVB membrane with the outer plasma membrane.

To date, at least three WNT signaling pathways have been characterized: the canonical WNT pathway stabilizing beta‐catenin, the non‐canonical, beta‐catenin‐independent planar cell polarity (PCP) pathway, and the non‐canonical WNT/calcium pathway [18]. Binding of a canonical WNT ligand, such as WNT3a, to its receptor Frizzled (Fzd) leads to beta‐catenin stabilization and the transcription of target genes [8, 19]. Binding of a non‐canonical ligand, such as WNT5a, to Fzd triggers recruitment of non‐canonical co‐receptors including ROR1/2, RYK, or RTK, and induces various cellular outcomes, such as cytoskeletal changes or modulating cellular calcium levels [19, 20].

Altered expression and dysregulated signaling through the non‐canonical WNT5a ligand has been linked to various cancers. Interestingly, WNT5a is overexpressed in a subset of cancers, such as melanoma [21, 22, 23, 24], gastric [25], and pancreatic cancers [26], and, as a consequence, promotes tumor progression. By contrast, WNT5a is inactivated in other cancer types, such as hematological malignancies [27, 28] and colorectal carcinoma [29], suggesting tumor‐suppressive functions [5, 30]. Bauer and colleagues proposed that the different functions of WNT5a are due to the production of two distinct protein isoforms, WNT5a Long (WNT5aL) and WNT5a Short (WNT5aS), originating from alternative transcriptional starting sites [5]. The WNT5aL transcript initiates at exon 1 (predicted to yield a 380 amino‐acid precursor protein). The two other transcripts start 718 and 578 nucleotides upstream of exon 2, resulting in an alternative exon 1β and a WNT5aS precursor of only 365 or 360 amino acids, thus lacking the first 15 or 20 N‐terminal amino acids of WNT5aL [5]. *In‐silico* modeling and experimental investigation revealed distinct signal peptide cleavage sites for both WNT5a isoforms, resulting in distinct secreted proteins [5]. While the stability, hydrophobicity, and canonical WNT signaling activity of the isolated proteins appeared to be comparable, they showed different effects on cell proliferation: WNT5aL inhibited and WNT5aS stimulated cancer cell proliferation [5].

Like other WNT ligands, WNT5a has been found on EVs, *e.g*., in the tumor microenvironment mediating invasiveness [14, 31] and activating profibrotic WNT signaling during cardiac fibrosis [10]. However, we still do not fully understand the mechanism and quantitative ratios of WNT5a secretion and transport into the extracellular space and onto the different EV fractions**—**neither *in vivo* nor *in vitro*.

To assess WNT transport quantitatively, we created fusion proteins of the two WNT5a isoforms, WNT5aL and WNT5aS, with the mScarlet and mOxNeonGreen fluorescent proteins (FPs). To test whether this strategy for fluorescent tagging jeopardized the stability and functionality of the target protein, we validated the functionality of all WNT5a fusion proteins. We confirmed their ability to block canonical WNT signaling in a TCF4/WNT luciferase assay and to induce invasiveness in the modified Boyden chamber. Since we did not detect functional differences between the short and long isoforms *in vitro*, we further focused on the commonly used isoform WNT5aL. In quantitative biophysical studies, we found that the mScarlet‐WNT5aL is either transported in the cell medium as a small, fast‐diffusing entity or on two different EV fractions, suggesting distinct mechanisms. All three fractions were characterized in detail by using fluorescence correlation spectroscopy (FCS) [32], fluorescence intensity distribution analysis (FIDA) [33], optical spectroscopy, and dynamic light scattering (DLS) to determine the sizes of the WNT5aL‐carrying particles and the number of WNT ligands per carrier. These findings contribute to our understanding of WNT transport dynamics and the broader complexity of EV heterogeneity and cargo distribution.

## Results

### Generation of fluorescently labeled WNT5a

**Fig. 1:**
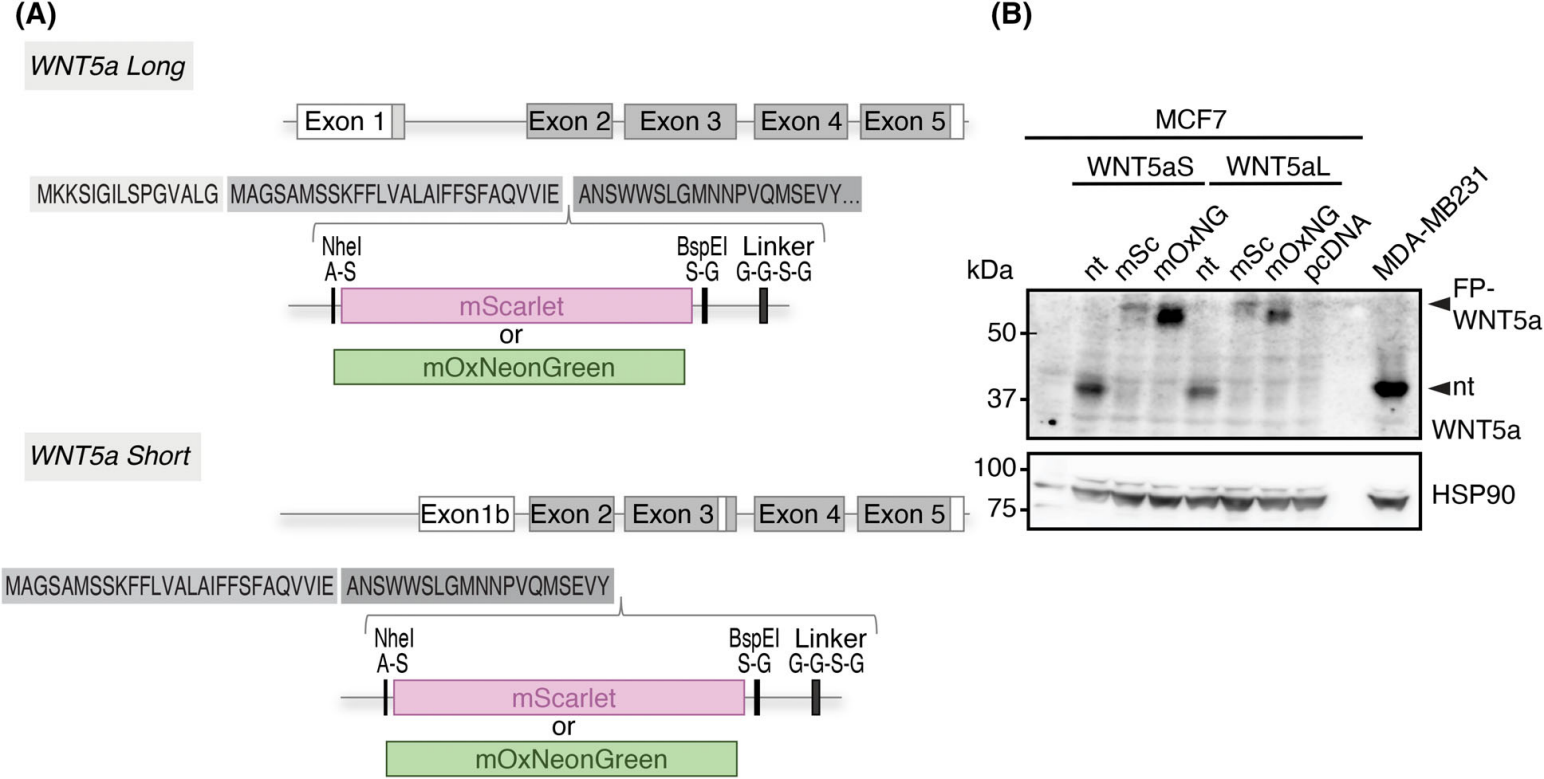
Fluorescent tagging of WNT5a isoforms. (A) Schematic presentation of the WNT5a isoforms, WNT5a Short (WNT5aS) and WNT5a Long (WNT5aL), and their tagging strategies. Fluorophores (FPs) were inserted after the predicted cleavage site of the signal peptide for both WNT5a isoforms, followed by a flexible linker. Restriction sites were included to facilitate the exchange of the FP. (B) Western blot analysis of the fusion constructs. All constructs were expressed in MCF7 cells and selected with G418. Cell lysate of MDA‐MB231 cells serves as the WNT5a positive control. Cell lysates were used for immunoblots with the WNT5a antibody. Arrowheads indicate the fluorescently tagged and non‐tagged (nt) WNT5a protein. HSP90 serves as a loading control. One of three independent experiments is shown. kDa, kilodaltons.

To fluorescently label the WNT5a isoforms, we inserted the FP at the N‐terminus of WNT5a, immediately after the predicted signal peptide cleavage site [34], as previously reported for tagging of WNT3a [35, 36]. Additionally, we engineered restriction sites before and after the FP to facilitate testing of other FPs. A ((SG)GGSG)‐linker was inserted after the FP to provide flexibility and solubility [37]. The first two amino acids of the linker are encoded by the BspEI restriction site (Fig. 1A). In contrast to a previously described Flag‐GFP‐WNT3a fusion [35], we did not insert any affinity tags. WNT proteins contain 22–24 cysteine residues, establishing disulfide bonds to confer structural stability [38, 39, 40, 41]. Since tagging of WNT3a with eGFP reduced the ability of the fusion construct to activate canonical signaling, we chose monomeric, cysteine‐free FPs. We hypothesized that, by using the cysteine‐free mScarlet [42], or a cysteine‐free version of mNeonGreen [43], mOxNeonGreen, optimized for fluorescent tagging of proteins located in the secretory pathways and oxidizing acidic compartments [44], we preclude disulfide bond formation between the FP and WNT domains and, thus, improper folding of the polypeptide chain and/or reduced protein stability (Fig. 1A).

We then tested the expression and found that all fusion proteins were expressed in MCF7 breast cancer cells and detected by western blotting (Fig. 1B). Expression of FP‐WNT5a isoform fusions in HEK293T cells and secretion into the cell medium was confirmed by western blot analysis (Fig. S1). Thus, our tagging strategy produced FP‐WNT5A fusion proteins that are secreted into the supernatant.

### Fluorescently labeled WNT5a isoforms maintain activity

WNT5a can bind to various receptors in a cell‐ and context‐dependent manner and initiate a variety of biological outcomes (reviewed in [45]). While multiple reporter assays have been established to determine the activation of canonical WNT signaling, there is still no standard assay available to measure the activity of non‐canonical WNT signaling. Therefore, we have performed two independent assays to assess the functionality of the WNT5a fusion constructs. First, we measured the ability of WNT5a to inhibit canonical WNT3a activity and, second, to induce invasiveness of luminal A breast cancer cells in an *in vitro*, modified Boyden chamber assay.

PCP and the beta‐catenin signaling pathways act antagonistically due to the competition for effector proteins, such as the Dvl proteins and receptors [45, 46]. Even though recent evidence suggests that WNT5a might also activate canonical signaling in a PCP‐independent and tissue‐dependent manner [45], WNT5a is known to repress TCF‐mediated WNT signaling, *e.g*., in hepatocellular carcinoma cells [47]. Accordingly, we probed the ability of our non‐canonical WNT constructs to inhibit canonical TCF4/WNT luciferase reporter activity in HEK293T cells as a readout for non‐canonical WNT activity.

**Fig. 2:**
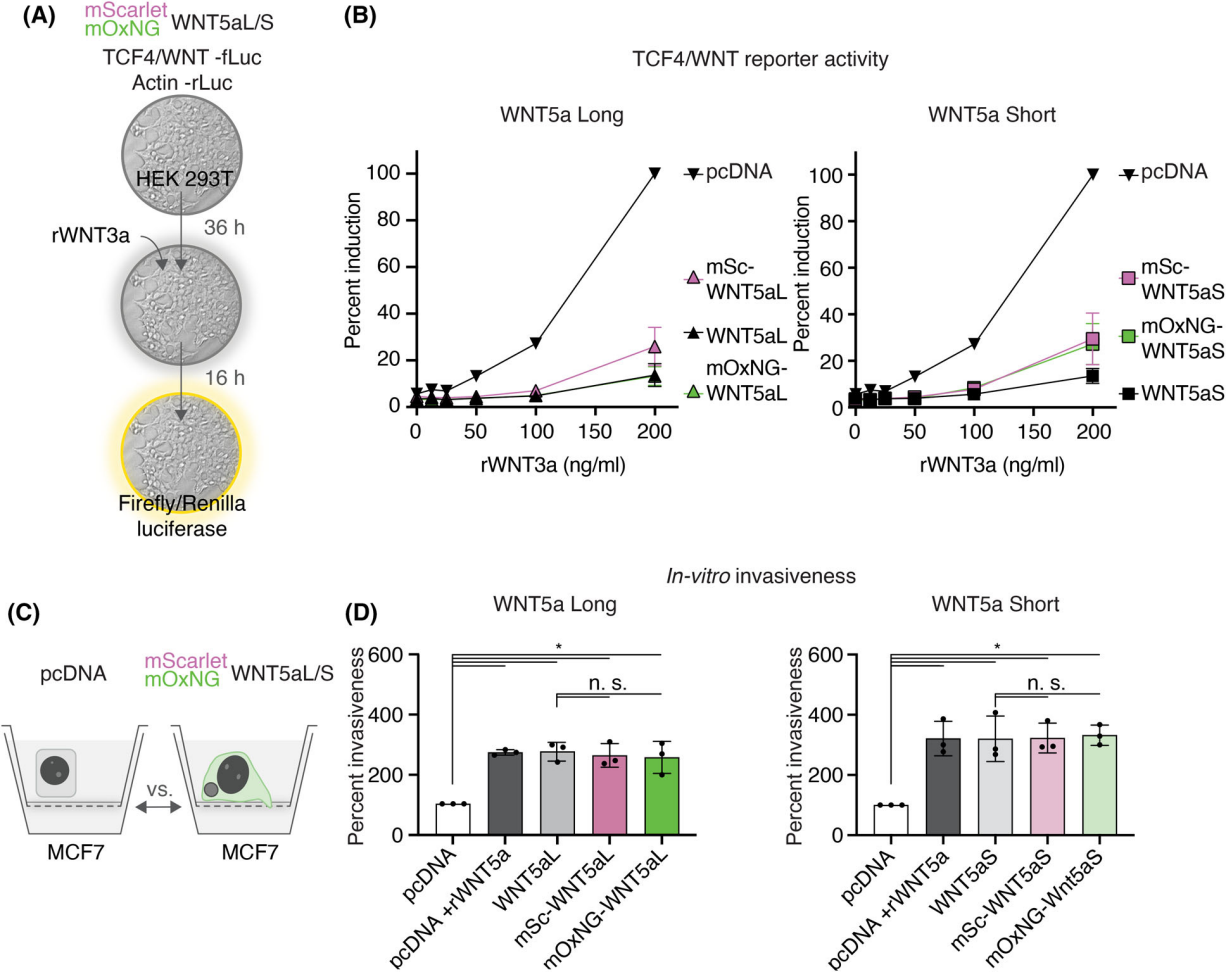
Fluorescently labeled WNT5a is functional in non‐canonical WNT assays. (A) Schematic presentation of non‐canonical TCF4/WNT luciferase assay. HEK293T cells were transfected with TCF4/WNT firefly and Actin‐renilla luciferase and the indicated WNT5a and pcDNA (control) plasmids. After 36 h, cells were stimulated by adding recombinant WNT3a (rWNT3a) with the indicated concentrations. The firefly/renilla luciferase signal was measured after 16 h. (B) WNT5a Short (WNT5aS) and WNT5a Long (WNT5aL) inhibit WNT3a‐mediated, concentration‐dependent luciferase induction in HEK293T cells. WNT reporter activity was determined as described in (A). Induced reporter activity was normalized to HEK293T pcDNA cells stimulated with recombinant WNT3a at a final concentration of 200 ng·mL^−1^. The graph represents the mean ± SEM of three independent experiments. (C, D) WNT5aL and WNT5aS induce the invasiveness of the luminal A breast cancer cell line MCF7. (C) Schematic presentation. MCF7 cells were transfected with the indicated plasmids and seeded into the modified Boyden chamber. Stimulation with recombinant WNT5a (rWNT5a) served as a positive control [48]. 96 h after seeding, the invasive capacity was measured by determining the number of cells that had penetrated the extracellular matrix. (D) Relative invasiveness (in percent) was determined by normalization to cells transfected with pcDNA. The graph represents the mean ± SEM of three independent experiments represented as dots. Two‐tailed Student's *t*‐test *P ≤ 0.05.

To measure the inhibitory potential on WNT3a‐mediated transcription, we expressed the respective plasmid‐encoded WNT5a (or pcDNA control plasmids) together with TCF4/WNT firefly luciferase and actin/renilla luciferase reporter plasmids in HEK293T cells. After 36 h, *i.e*., 16 h before reporter readout, the cells were stimulated by adding recombinant WNT3a (Fig. 2A). In control cells (pcDNA), WNT3a addition resulted in the expected concentration‐dependent activation of WNT reporter activity, whereas WNT reporter activity was suppressed in cells expressing WNT5aL or WNT5aS. Comparison of the untagged and mScarlet‐ or mOxNeonGreen‐tagged isoforms indicated that the FP domains had no significant impact on the observed inhibitory potential of WNT5a (Fig. 2B).

Next, we measured the induction of invasiveness in a modified Boyden chamber, as previously reported [48] (Fig. 2C). Expression of WNT5aL and WNT5aS induced invasiveness of the luminal A breast cancer cell line MCF7, similar to the treatment with recombinant WNT5a (Fig. 2D). These results demonstrate that the presence of the FPs did not influence the invasive potential of WNT5a in this assay (Fig. 2D).

### Both WNT5a isoforms, WNT5aL and WNT5aS, are transported via two distinct EV fractions

**Fig. 3:**
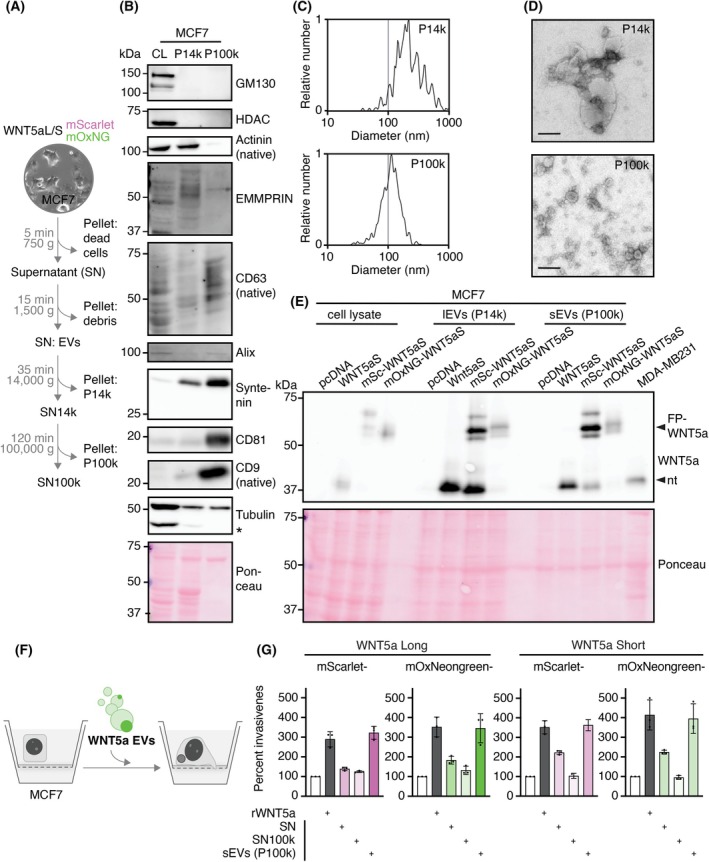
WNT5a is exported onto two types of extracellular vesicles (EVs) and induces invasiveness. (A) Schematic presentation of EV isolation by differential centrifugation. EVs were pelleted at 14 000 ***g*** (P14k) and 100 000 ***g*** (P100k). (B) Cell lysate (CL) and the pellets P14k and P100k were analyzed by western blot with the indicated antibodies, demonstrating distinct characteristics of both fractions. Asterisk marks a non‐specific band. Representative western blot of more than three independent experiments. kDa, kilodaltons. (C) Nanoparticle tracking analysis (NTA, ZetaVIEW®) identified P14k as large EVs (100–1000 nm, lEVs) and P100k as small EVs (~100 nm, sEVs). Measurements were performed on four independent isolations of lEvs and sEVs from MCF7 cells; representative EV measurements of EVs from mScarlet‐WNT5aShort expressing MCF7 cells are shown. (D) Representative electron microscopy images of P14k and P100k resuspended in PBS. Images were taken from EV preparations from three different days from mScarlet‐WNT5aS expressing MCF7 cells. Scale bar = 100 nm. (E) Western blot analysis of the cell lysate (CL) and the pellets P14k (lEVs) and P100k (sEVs) confirms the export of the WNT5a constructs onto both EV fractions. Blot for WNT5a Short (WNT5aS), for WNT5a Long (WNT5aL), see Fig. S2. Arrowheads indicate the fluorescently tagged and non‐tagged (nt) WNT5a protein. Cell lysate of MDA‐MB231 cells serves as a WNT5a positive control. FP, fluorescent protein; mSc, mScarlet; mOxNG, mOxNeonGreen. Due to high EV yields needed to perform western blot analysis, western blots of FP‐WNT5a‐carrying EVs (Fig. 3D; Fig. S2) were performed once for all FP‐WNT5aS and twice for all FP‐WNT5aL constructs. Their presence on EVs was confirmed in subsequent experiments employing complementary methodologies, *e.g*., fluorescence spectroscopy and FCS. (F) Scheme of the modified Boyden chamber assay. (G) The different fractions of the cell culture supernatant (SN) of MCF7 cells expressing the indicated WNT5a constructs or recombinant WNT5a (rWNT5a, 100 ng·mL^−1^, served as positive control) were added to the Boyden chamber to measure the induction of invasiveness. For sEVs, 1 μg of EV protein (quantified via Lowry Assay) was added. In conditions stimulated with SN, equivalent volumes to those added in the EV condition were utilized. Relative invasiveness (in percent) was determined by relating the number of invasive cells of the treated conditions to the untreated control. The graph represents the mean ± SEM of three independent experiments shown by dots.

To probe whether WNT5aL and WNT5aS are loaded onto EVs for transport, we used differential centrifugation to isolate different EV fractions [14, 49]. After centrifugation at 14 000 ***g*** for 35 min, we harvested the P14k pellet. By ultracentrifugation of the supernatant (SN14k) at 100 000 ***g*** for 2 h, we isolated the P100k pellet (Fig. 3A). Both pellets were resuspended in PBS and analyzed by western blotting. As shown in Fig. 3B, the P14k fraction was enriched in EMMPRIN and the cytoskeletal protein actinin [50]. These markers were essentially absent in the P100k fraction; instead, the tetraspanins CD9, CD63, and CD81 were enriched (Fig. 3B). GM130 and HDAC, markers for the Golgi apparatus and nucleus, respectively, were absent in both pellets. Next, we performed nanoparticle tracking analysis (NTA) with the ZetaVIEW® instrument to obtain information on the average size of the EVs. As shown in Fig. 3C, the P14k particles had diameters between 100 and 1000 nm, while the peak of the P100k EVs was at ~100 nm diameter (Fig. 3C). Electron microscopy revealed distinct size and morphology of the two particle fractions P14k and P100k (Fig. 3D). In summary, differential centrifugation enabled the isolation of two distinct EV fractions, P14k (large EVs) and P100k (small EVs) (Fig. 3A–D).

We chose EVs secreted by MCF7 cells for the functional analyses described below since EVs from malignant cells have the potential to induce invasiveness in the modified Boyden chamber, in contrast to EVs from benign cells [50]. First, we performed differential centrifugation of the cell culture supernatants harvested from MCF7 cells expressing WNT5aL, WNT5aS, and the corresponding fluorescently tagged constructs (Fig. 3E; Fig. S2), and then analyzed them by western blotting. Both WNT5a isoforms and their tagged constructs were detected in both EV fractions. It is noteworthy that the WNT5a fusions also gave rise to a band at ~37 kDa, *i.e*., at the size expected for non‐tagged WNT5a. Since MCF7 cells do not harbor endogenous WNT5a, some of the FPs were apparently cleaved off (Fig. 3E). This was also observed when eGFP‐tagged WNT3a was analyzed by western blotting by Wesslowski and colleagues [36].

To measure the *in vitro* invasiveness of MCF7 cells, we incubated MCF7 cells either with recombinant WNT5a (gene ID 7474 (human), no information on the isoform given by the vendor), the unfractionated cell culture supernatant, isolated small EVs (P100k fraction), and the EV‐free supernatant (SN100k) of WNT5a‐expressing MCF7 cells (Fig. 3F,G). Interestingly, the P100k fraction of both WNT5a isoforms induced invasiveness in the modified Boyden chamber (Fig. 3G).

### Large EVs carry more WNT5aL proteins than small EVs

For quantification of the number of WNT5a ligands loaded onto sEVs and lEVs, we selected the mScarlet‐WNT5aL fusion protein, taking advantage of its fluorescence in the red spectral range, which allows for measurements with minimal autofluorescence background. Of note, we did not find differences in secretion by immunoblotting or functional differences in the applied assays between mScarlet and mOxNeonGreen. The construct was expressed in HEK293T cells, a cell line frequently used for WNT assays and production of WNT‐conditioned medium (CM). The CM harvested from the HEK293T cells was fractionated into lEV (P14k), sEV (P100k), and supernatant (SN100k) samples as described for the MCF7 cells, and the relative amounts of mScarlet‐WNT5aL in the supernatant and the EV fractions were estimated by fluorescence spectroscopy. Emission spectra of all three fractions show the characteristic band of mScarlet centered on 594 nm (Fig. S3A). From the spectral areas, we estimate that at least 90% of the mScarlet‐WNT5aL fusion proteins are in the supernatant fraction (SN100k) and less than 10% in the EVs fractions.

For quantification of the number of mScarlet‐WNT5aL molecules associated with each of the EVs, we took advantage of FIDA, a type of fluorescence fluctuation spectroscopy that allows us to extract the concentration and brightness values of multiple species diffusing in solution, which is based on fluorescence intensity measurements with a confocal microscope [33]. For FIDA, we counted the number of photons emitted within a 30‐μs interval from a small observation volume (typically, 10^−15^ L = 1 μm^3^) placed into the sample. This measurement was repeated several million‐fold by raster‐scanning the observation volume through the solution, resulting in a large number of confocal images. After cluster analysis to remove larger aggregates, the data were compiled in photon counting histograms (PCHs) displaying the number of measured events (*i.e*., pixels) with a particular photon count as a function of the photon count. The experimentally determined PCHs form the basis for modeling with the FIDA method [33].

**Fig. 4:**
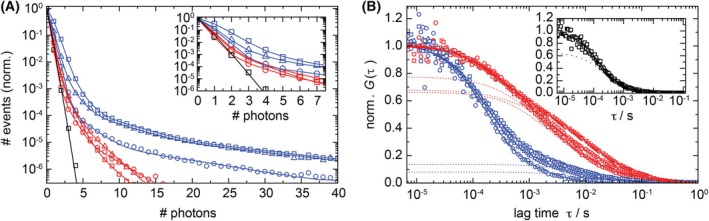
Fluorescence intensity distribution analysis (FIDA) and fluorescence correlation spectroscopy (FCS) on mScarlet‐WNT5aL‐carrying small EVs (sEV) and large (lEV) samples isolated from conditioned medium (CM). (A) Photon counting histograms (PCHs) of sEV (red symbols) and lEV (blue symbols) samples and model fits (lines). Data were taken on three independent samples each (samples 1–3, shown using circles, triangles and squares). The reference PCH of mScarlet (black) is included for comparison. Inset: expanded view of the PCHs for photon counts below eight. (B) Experimental FCS autocorrelation curves measured on sEV (red symbols) and lEV (blue symbols) samples; solid lines, model fits according to Eq. 2; dotted lines, contribution of the EVs (*i.e*., fast component with a single mScarlet‐WNT5aL subtracted). Data were taken on three independent samples each (samples A–C, shown using circles, triangles and squares). Note that we cannot detect a significant vesicle fraction in lEV sample A; therefore, the autocorrelation function is due to the fast‐diffusing species only. Presumably, the lEVs were aggregated and, consequently, rejected in the selection of FCS traces for analysis. The experimental data are averages over 67, 43, and 49 (out of 150 for sEV samples A, B and C, respectively) and 103, 84, and 55 (out of 120 for lEV samples A, B and C, respectively) selected 10‐s curves. Inset: FCS curve of purified mScarlet (symbols, recorded with 2 μW of 560‐nm laser light); solid line, single‐component fit with Eq. 2; dotted line, diffusional correlation part only.

For all EV‐containing samples, the PCHs show a huge peak of events in the range below five photon counts (Fig. 4A). Comparison with a sample containing purified mScarlet protein assigns this feature unambiguously to the presence of a diffusing entity that contains only a single mScarlet‐WNT5aL. Notably, this cannot be the bare fusion protein, as the palmitoyl moiety of WNT5a [4, 51, 52] must always be shielded from the aqueous medium by an amphiphilic moiety to ensure solubility. Furthermore, the PCHs feature broad tails toward higher numbers of photons arising from the presence of a small fraction of bright objects. For lEV samples, this tail extends to large photon numbers, indicating very bright particles carrying large numbers of fluorophores.

For quantitative analysis, we adopted an approach from our previous work on ligand binding to endothelin receptors on virus‐like particles [53]. We introduced a heuristic multi‐component model to represent, on the one hand, the monomeric (single mScarlet‐WNT5aL) fraction and, on the other hand, several EV populations with distinct brightness values describing the broad tails of the PCHs. In FIDA, this model is fitted to the experimental PCH data (Fig. 4A) to yield the concentrations and brightness values of the components (Table S1). Our analysis reveals that the monomeric fraction is in the nanomolar range for all samples, whereas EVs are less abundant by about 2–3 orders of magnitude. Nevertheless, EVs contribute significantly to the total fluorescence because, on average, 56 ± 3 and 64 ± 2 fluorescent mScarlet‐WNT5aL proteins reside on each of the sEVs and lEVs, respectively (Table S1). Here and below, we quote uncertainties as provided by the fitting routine (plus error propagation for computing averages). A closer look at the FIDA parameters in Table S1 shows that component 2 is the predominant EV fraction in the (concentration‐weighted) average. Notably, the real number of WNT5aL proteins on EVs is almost twice as large as these averages because only 52 ± 1% of the mScarlet proteins carry an active fluorophore (see Materials and methods section). In addition to the average numbers of fluorescent mScarlet‐WNT5aL proteins, we have calculated standard deviations (SDs) as measures of the widths of the distributions (Table S1). The SDs are large, 32.2 ± 6.3 and 100.5 ± 8.0 for sEVs and lEVs, respectively, indicating broad number distributions, as already anticipated from the PCH data in Fig. 4A.

Next, we determined the average sizes (hydrodynamic diameters) of the EVs in our samples by using DLS with intensity distribution analysis. Exemplary data are shown in Fig. S3B. For sEVs and lEVs, we obtained 93 ± 26 nm and 395 ± 65 nm (mean ± SEM, *n* = 3), respectively. These sizes, however, include contributions from all EV species in the sample and not only from those carrying mScarlet‐WNT5aL proteins. Therefore, we turned to FCS, a confocal microscopy‐based method for size determination of fluorescent colloidal nanoparticles in solution via Brownian diffusion [54, 55]. While this method is simple and straightforward to use with pure samples of a single diffusing entity, the presence of multiple diffusing species with a wide range of sizes and concentrations, as implied by our FIDA results, presents a formidable challenge. We also note that faithful FCS size determination is limited to particle diameters markedly smaller than the focus extension of our confocal microscope (~600 nm).

For FCS analysis, we placed the observation volume into the sEV and lEV samples and took intensity‐time traces of the fluorescence emission over 1500 and 1200 s, respectively. The data were split into 10‐s segments, which were individually autocorrelated to identify and remove segments featuring large spikes (see Materials and methods and Fig. S4), which are due to infrequently appearing very large and bright particles, including inevitably present aggregates. The averages over the remaining autocorrelation curves (Fig. 4B) display a distinctly different temporal decay between sEV and lEV samples. For the lEV samples, there is an early decay of the correlations followed by a well‐separated second step at longer times. By contrast, the correlations of the sEV samples drop gradually in time. Within the two data sets, the curves are qualitatively similar, and variations suggest the presence of different amounts of EVs in the samples.

These observations were confirmed by quantitative analysis based on a model function with multiple species (Eq. 2, Materials and methods). By fitting the model to the experimental autocorrelation functions, we extracted the relative number fractions (concentrations) as well as the diffusional correlation times. The detailed strategy is described in Materials and methods; all parameters and best‐fit values are compiled in Table S2. A fit with two components describes the experimental autocorrelation functions of the lEV samples very well. The predominant fraction is a fast‐diffusing monomeric entity with a diffusional correlation time, $\tau_{D}$ = 0.43 ms, corresponding to a hydrodynamic diameter, 2*R*_*H*_ = 10.0 ± 0.8 nm. The second, slowly diffusing lEV component is only a minority fraction. It is less abundant by orders of magnitude but nevertheless well visible in the autocorrelation curves because of its high brightness (~64‐fold enhanced, as determined by FIDA). Notably, in FCS theory, the two components are weighted with their squared brightness in the autocorrelation (Eq. 2). Consequently, the lEV correlations are amplified ~4000‐fold over the monomeric fraction, making the experiment very sensitive to these particles. Diffusion of the lEV component is described by a correlation time, $\tau_{D}$ = 10.4 ± 1.5 ms, from which Eq. 3 yields a diameter, 2*R*_*H*_ = 240 ± 20 nm (averages over three samples).

Modeling the autocorrelation functions of the sEV samples is more involved. There is again the fast correlation decay (τ < 1 ms), as was observed for the lEV samples. However, the more slowly diffusing fraction cannot be modeled with a single EV species, and close inspection of the fit shows systematic variations at larger correlation times (τ > 1 ms) for all sEV data sets, clearly indicating a marked size dispersion of the sEV fraction. Therefore, we introduced two diffusing sEV components into our fit model, with correlation times $\tau_{D,2}$ and $\tau_{D,3}$ (Table S2), and then computed the overall correlation time as the population‐weighted average. Using Eq. 3, we determined the diffusion coefficients, averaged them over the three samples and finally arrived at an overall average diameter, 2*R*_*H*_ = 122 ± 22 nm.

## Discussion

In this work, we examined the biophysical properties of functionally validated WNT5a proteins on distinct EV populations for the first time. By applying sophisticated fluorescence fluctuation techniques, FIDA and FCS, we quantitatively characterized transport vehicles in the extracellular fluid of WNT5a, a protein with diverse roles and implications in diseases such as cancer. Our findings contribute to a better understanding of WNT signaling and the role of EVs in this process.

Previous studies on the canonical WNT3a ligand reported that fusion with an FP marker protein results in partial loss of function, as inferred from activity assessments with transcriptional reporter assays or beta‐catenin stabilization [35, 36]. As WNT5a can induce multiple signaling outcomes, some of which are independent of transcriptional activation, testing the functionality of tagged non‐canonical WNT ligands was crucial for this study.

By using the modified Boyden chamber [48], we analyzed the pro‐invasive phenotype of tagged and non‐tagged WNT5a variants. Interestingly, WNT5aS and WNT5aL induced similar invasiveness in this *in vitro* assay. This might appear to contradict the results of Bauer *et al*. [5], who showed an induction of proliferation by WNT5aS and an inhibition of proliferation by WNT5aL. While they found WNT5aL frequently downregulated in tumors, WNT5aS was usually upregulated in tumors such as breast cancer. Hence, they concluded that WNT5aL and WNT5aS have varying and distinct activities in cancer. Since tumor cell proliferation and invasion are distinct biological processes [56], a pro‐invasive potential of both isoforms in an *in vitro* assay does not exclude distinct proliferative capacities in cancer *in vivo*, however. It remains to be resolved if distinct yet not sufficiently explored mechanisms of secretion are responsible for the suggested different effects of WNT5a Long and Short [5], or if the information mediating distinct receptor binding and affinity is encoded within the initial regions of these proteins. In our assay, both isoforms were pro‐invasive and, importantly, without effects of FP tagging. While it is known that EVs from MCF7 cells transfected with control plasmids can moderately stimulate *in vitro* invasiveness [57], the exact mechanism of increased induction of invasiveness mediated by EVs from WNT5a overexpressing MCF7 cells—whether through, *e.g*., ligand‐receptor interactions or altered EV cargo—remains to be elucidated.

PCP and beta‐catenin signaling pathways are known to interact antagonistically, *e.g*., non‐canonical WNT5a has been shown to antagonize TCF‐mediated transcription [47]. This crosstalk might be caused by competition for cytoplasmic effector proteins, or at the ligand–receptor level [46]. WNT5a binding to ROR2 can inhibit canonical WNT signaling [58], and WNT5a can block WNT3a binding to Fzd2 by competition [59]. However, WNT5a has also been shown to activate canonical signaling in a context‐ and concentration‐dependent manner [58, 60, 61, 62]. In our assays, both WNT5a isoforms inhibited the induction of TCF4‐mediated transcription by recombinant WNT3a in HEK293T cells; fluorescent tagging had no interfering effect. However, we did not distinguish at which level this antagonism occurs (*e.g*., at the receptor level or at the level of effector proteins such as Dvl).

Overall, FP tagging did not interfere with the function of non‐canonical WNT5a, different from the findings on WNT3a presented in previous studies [35, 36]. We modified the tagging strategy with respect to the previously published WNT3a by using cysteine‐free FPs and another linker sequence. Because we applied functional assays different from earlier work, a direct comparison remains elusive, however.

Could overexpression of WNT5a *per se* modulate the formation of and transport onto EVs? A genome‐wide RNAi and CRISPR/Cas9 screen targeting WNT signaling and trafficking genes identified numerous mediators of EV secretion, such as ALIX and YKT6 [63]. WNT‐mediated GSK3 inactivation might suppress Rab27, thereby modulating EV release [63, 64]. Non‐canonical WNT signaling can influence cytoskeletal rearrangements, cell motility, invasiveness, and EV release factors such as Rabs, Rho GTPases, calcium, and ARF6 with potentially direct and indirect effects on EV release (as reviewed in [65]). Moreover, WNT5A has been proposed to control calcium‐dependent exosome release in malignant melanoma cells and through WNT5a/PI3K/miR‐122 in hepatocyte differentiation [66, 67]. Further studies addressing the effects of WNT overexpression on EV release are necessary to fully understand the mechanisms and fractions of WNT5a carriers in the CM of WNT‐expressing cells.

Nanoparticles can be characterized using various techniques for determining their physical, chemical, and biological properties. Well‐established characterization methods include DLS, transmission electron microscopy (TEM), and NTA. Here we have combined advanced fluorescence fluctuation techniques, FIDA and FCS, providing a new level of resolution in the molecular characterization of single EVs. While these techniques are well established in biophysical research, their application to EV characterization is novel and innovative. Moreover, in combination with fluorescent protein fusions, they enable us to selectively investigate EVs carrying the proteins of interest. FIDA revealed >100 and >120 WNT5a proteins associated with sEV and lEV particles, respectively, whereas FCS yielded average hydrodynamic radii of ~120 and ~240 nm. Notably, larger lEVs in the ensemble are suppressed, as our FCS implementation reaches its size limit.

Interestingly, we found that, in the CM of HEK293T cells, the major fraction of WNT5a was associated with (non‐EV) carriers of ~10 nm diameter containing a single mScarlet‐WNT5a fusion protein. Data on the secretion and transport of non‐canonical WNTs are scarce, but earlier studies investigated WNT3a diffusing in the supernatant of secreting cells [4, 35]. Large WNT complexes in serum‐free medium [68] were considered inactive, whereas monomeric WNT3a in the presence of a detergent (CHAPS) was found to be active [4]. While mEGFP‐WNT3a from stably expressing L cells was reported to exist as signaling‐competent, self‐associating homo‐trimers shielding the lipid moiety of the WNT3a proteins [35], which were disassembled by interaction with the extracellular domain of Fzd8 or secreted frizzled‐related protein 2 (sFRP2) [35].

With SDS/PAGE, we detected a band located at the size of unlabeled WNT5a in the MCF7 EV fractions after sample preparation (Fig. 3E), consistent with findings by Wesslowski *et al*. [36]. Presumably, some of the fusion constructs are partially cleaved during SDS/PAGE preparation. However, the predominant component containing a single fluorescent moiety found in the FCS and FIDA data cannot be assigned to a cleaved‐off mScarlet protein because it diffused significantly more slowly than mScarlet alone, containing a (non‐EV) single mScarlet‐WNT5a bound to a yet unknown carrier of ~10 nm diameter.

While we have characterized the transport of WNT5a ligands on different carriers in the CM of HEK293T cells, the distinct cellular origin of the EVs or their secretory mechanisms have remained elusive. The abundant, fast‐diffusing (non‐EV) WNT5a proteins could bind to EVs after secretion and be part of a protein corona, which has been described for synthetic nanoparticles [69, 70] and EVs in the bloodstream [71, 72]. Menck *et al*. [14] demonstrated that recombinant WNT5a can associate with vesicle membranes both specifically and non‐specifically. After exposing WNT5a‐negative tumor vesicles to high concentrations of recombinant WNT5a, a significant portion of the protein attached to distinct EV fractions, with some remaining in the supernatant, indicating weaker, non‐specific binding. However, they also identified a specific mechanism for the export of vesicle‐associated WNT5a involving the WNT cargo receptor Evenness Interrupted (Evi)/Wntless (Wls) [14]. For the transport and release of WNT ligands from this cargo receptor, different mechanisms have been proposed (as reviewed in [73]): separation at the plasma membrane or remaining together on vesicles [74]. Studies in Drosophila suggest the latter, with an Evi/Wls‐WNT complex facilitating direct handover to receptors. Alternatively, WNTs might separate from Evi/Wls at the plasma membrane, possibly involving lipid‐binding carrier proteins and glypicans, influenced by pH changes, or after re‐endocytosis from the plasma membrane [75, 76]. The FIDA and FCS studies presented here provide clear evidence of more than 100 WNT5a proteins bound to EVs under conditions of picomolar and nanomolar concentrations of EVs and small WNT5a carriers, respectively. The presence of these stable entities at very low concentrations implies strong binding of the WNTs to these carriers. Interestingly, both EV fractions, P14k and P100k, contained a rapidly diffusing component—in view of the particle size (~10 nm diameter) presumably non‐EV in nature—which may reflect sample purity limitations inherent to the technical characteristics of the method, such as residual SN components despite PBS washing. However, the possibility of a secondary, artifact‐driven formation of this fraction cannot be excluded. The precise mechanisms underlying EV association and subsequent transfer to WNT receptors remain to be elucidated.

Despite certain limitations of studies involving overexpressed proteins, we gained valuable information on WNT5a transport in the CM of HEK293T cells. In our study, most WNT5a remained in the EV‐free SN100k after ultracentrifugation, while only a small fraction of WNT5aL was transported via EVs secreted by HEK293T cells. For the first time, we quantitatively compared the association of WNT5aL with two distinct EV fractions. The average number of WNT5aL proteins was similar (only 15% larger) on lEVs than on sEVs; therefore, the average surface density of WNT5aL was smaller for lEVs.

To conclude, our results will be valuable for researchers applying WNT‐conditioned media in biological assays and mechanistic studies. Quantitative studies of other WNT ligands in other (cellular) contexts and comparisons with our current results will provide further insights into the relative proportions of different WNT transport pathways in the signaling process.

## Materials and methods

### Cell culture

HEK293T (RRID:CVCL_0063) and MCF7 (MCF‐7, RRID:CVCL_0031) cells were purchased from the ATCC (Manassas, VA, USA). Authentication was performed using single‐nucleotide polymorphisms profiling (Multiplexion, Heidelberg, Germany). HEK293T cells were grown in DMEM, and MCF7 cells in RPMI (+ glutamine) (both Thermo Fisher Scientific (Waltham, MA, USA)). Media were supplemented with 10% fetal bovine serum (FBS, Biochrom, Berlin, Germany) without antibiotics. Mycoplasma contamination was ruled out on a regular basis.

### Cloning of tagged WNT5a constructs

All constructs were generated using In‐Fusion cloning (Takara Bio, Kusatsu, Japan) according to the manufacturer's protocol. Tagged WNT5a constructs of the two isoforms WNT5aL and WNT5aS [5] were cloned into a pcDNA3.2/V5‐DEST (STOP before V5) vector by insertion of the FP and a C‐terminal flexible (G‐G‐S‐G)‐Linker after the predicted signal peptide cleavage site [34, 77]. The SignalIP server (version 4.1) utilizes neural networks trained on sets of prokaryotic and eukaryotic protein sequences to predict signal peptides and their cleavage sites [34, 77]. Inserts were PCR‐amplified from pcDNA‐WNT5a (Addgene #35911) and gBlocks containing the respective FPs. For the WNT5aS constructs, the signal peptide was modified accordingly in the PCR step. Sanger sequencing of the whole constructs was performed for quality control (GATC/Eurofins Genomics). Oligonucleotides are listed in Table S3, and plasmids in Table S4.

### Plasmid transfection

Plasmid transfection was carried out using the TransIT LT1 Transfection Reagent (Mirus Bio, Madison, WI, USA) or FuGENE® HD Transfection Reagent from Promega (Madison, WI, USA) following the manufacturer's protocol.

### TCF4/WNT‐luciferase assay

WNT signaling activation was measured using the TCF4/WNT luciferase assay on HEK293T cells as previously described [78]. In short, 7500 cells per well were seeded in DMEM supplemented with 10% FBS into 384‐well white, flat‐bottom polystyrene plates (Greiner, Mannheim, Germany). After 24 h, cell transfection was performed with 20 ng of TCF4/WNT firefly luciferase reporter, 10 ng of actin–Renilla luciferase reporter, and 20 ng of the respective WNT or control (pcDNA3.2/V5‐DEST) plasmids using the TransIT LT1 Transfection Reagent (Mirus Bio, Madison, WI, USA). The transfected DNA was equalized with the control plasmid (pcDNA3.2/V5‐DEST).

For induction of WNT3a signaling, recombinant mouse WNT3a was added (PeproTech, Hamburg, Germany, see Table S5) to a final concentration of 100 ng.mL^‐1^ 16 h before luciferase readout.

48 h after transfection, luminescence was measured using the plate reader Mithras LB940 (Berthold Technologies, Bad Wildbad, Germany). The TCF4/WNT luciferase signal was adjusted by normalizing it to the actin‐Renilla signal of each well before further data analysis.

### Immunoblot analysis of cell lysate and secreted WNT proteins

Whole‐cell protein extraction was performed using the commercially available RIPA lysis buffer (Thermo Fisher Scientific, Waltham, MA, USA), supplemented with HALT™ protease and phosphatase inhibitor cocktail (Thermo Fisher Scientific). Pierce BCA Protein Assays (Thermo Fisher Scientific) were performed to determine protein concentrations.

Secreted WNTs were analyzed using the Blue Sepharose pull‐down [79]. In short, the CM was harvested 48–72 h after cell seeding or transfection. The supernatant was centrifuged at 2000 ***g*** for 5 min to deplete dead cells. Triton X‐100 was added to a final concentration of 1% (v/v). Blue Sepharose 6 Fast Flow beads (17–0948‐01; GE Healthcare) were washed 3 times with a washing buffer (150 mm KCl, 50 mm Tris–Cl, pH 7.5, 1% (v/v) Triton X‐100), added to the supernatant/triton and incubated at 4 °C overnight. On the next day, the beads were again washed twice (centrifugation at 2700 g for 5 min at 4 °C). The beads were resuspended in reducing 2× Laemmli buffer and boiled at 95 °C for 5 min before loading equal amounts of supernatant onto a gel for western blotting.

For whole cell lysates, 10–30 μg of protein were loaded onto pre‐cast Bolt™ Bis‐Tris Plus gels (4–12%; Life Technologies, Carlsbad, CA, USA) in MOPS buffer and then transferred to Amersham Protran 0.45 μm western blot nitrocellulose membranes (GE10600002; GE Healthcare, Waukesha, WI, USA). Antibodies are listed in Table S6, and western blot full scans are shown in Figs S5–S9.

### Fractionation of CM and isolation of extracellular vesicles

EVs were isolated by differential centrifugation (see Fig. 3A) as previously described [14]. Cells were cultured for up to 48 h in the respective medium supplemented with EV‐free, heat‐inactivated FBS. If not stated otherwise, the CM from six confluent T175 cell culture flasks was collected and pooled for one EV isolation. Supernatants were centrifuged at 750 ***g*** for 5 min and 1500 ***g*** for 15 min to remove cells and debris, respectively. Next, centrifugation was performed at 14 000 ***g*** for 35 min to pellet lEVs. For the isolation of sEVs, the supernatant was collected and filtered through a 0.2 μm sterile filter before the last centrifugation step at 100 000 ***g*** for 2 h. Ultracentrifugation was performed using the ultracentrifuge Optima™ L80M (Beckman Coulter) with the rotor SW32Ti (Beckman Coulter #369650) and polycarbonate ultracentrifugation tubes, also from Beckman Coulter (#355631). The pellets were washed once with PBS (w/o Mg^2+^/Ca^2+^) and then resuspended in 100–200 μL PBS or RIPA lysis buffer. The EV protein concentration was measured with the Lowry‐like DC Protein Assay from Bio‐Rad (Feldkirchen, Germany). EV samples were aliquoted and stored at −20 °C to avoid repeated freeze–thaw cycles. The EV‐free supernatant collected after ultracentrifugation was stored at 4 °C. All samples were used within 30 days or stored at −80 °C and, before use, thawed on ice. As indicated, western blot analysis of some EV fractions was performed under native conditions (non‐reducing Laemmli, no boiling). Due to high EV yields needed to perform western blot analysis, western blots of FP‐WNT5a‐carrying EVs (Fig. 3D; Fig. S2) were performed once for all FP‐WNT5aS and twice for all FP‐WNT5aL constructs. Their presence on EVs was confirmed in subsequent experiments employing complementary methodologies, *e.g*., fluorescence spectroscopy and FCS.

### Nanoparticle tracking analysis (NTA)

EV size distribution was analyzed using a ZetaVIEW® laser scattering microscope (ZetaVIEW® S/N 18–351, Camera 0.714 μm/px, Particle Metrix, Inning am Ammersee, Germany) according to the manufacturer's instructions. EV samples were diluted in PBS to obtain an average of 100–400 counted particles per frame. Particles were tracked in short videos of 1 s at app. 25 °C at 11 distinct positions. Particle concentration and size were analyzed using the ZetaVIEW® software 8.05.05 SP2. Analysis parameters: maximum area: 1000, minimum area: 10, minimum brightness: 30.

### Dynamic light scattering (DLS)

EV size distributions were obtained by intensity distribution analysis of light scattering data collected on a Zetasizer Nano‐ZS instrument (Malvern Instruments, Malvern, UK) equipped with a 633‐nm He–Ne laser.

### Electron microscopy

For electron microscopy (EM), P14k and P100k were resuspended in PBS and adsorbed onto glow‐discharged, carbon‐coated copper grids (300 mesh, Science Services, Munich, Germany), washed in water (Braun, Melsungen, Germany) and negatively stained with 2% aqueous uranyl acetate. Micrographs were taken with a Zeiss EM 910 at 80 kV (Carl Zeiss, Oberkochen, Germany) using a slow scan CCD camera (TRS, Moorenweis, Germany).

### Microinvasion assay

The modified Boyden chamber assay was used to measure *in vitro* cell invasion, as previously reported [48]. 10^5^ MCF7 cells were seeded in triplicates in EV‐free medium onto an ECM (3432‐005‐01; Cultrex PathClear Basement Membrane Extract, Bio‐Techne, Minneapolis, MN, USA) coated polycarbonate membrane (PC99CP81030, Pieper Filter, Bad Zwischenahn, Germany, pore diameter 10 μm) of a Boyden chamber (manufactured at the University Medical Centre of Göttingen). Cells were treated for 96 h with the respective fractions. Cell invasiveness was quantified by relating the invasive cell count from the lower wells to the cell count in the unstimulated control wells. See also Table S5 for treatments.

### Quantitative fluorescence microscopy on secreted WNT5a

EV‐containing samples were prepared from CM harvested from HEK293T cells, expressing the respective pcDNA3‐based mScarlet‐WNT5a constructs under selection pressure by using the antibiotic G418. A reference sample containing about 10 nm mScarlet FP was prepared by adding a few microliters of a concentrated stock solution of mScarlet (expressed in *E. coli*) to the supernatant fraction of CM harvested from HEK293T cells transfected with an empty pcDNA3 vector.

The sample holder was assembled from two coverslips (18 × 18 mm^2^, Hirschmann Laborgeräte, Eberstadt, Germany) that were briefly torched to remove fluorescent contaminants, incubated in MilliQ water supplemented with 1 mg·mL^−1^ BSA for 10 min, and then rinsed five times with sodium phosphate buffer (40 mm, pH 7.4) supplemented with NaCl (300 mm). For the measurements, 30 μL of the sample solution was kept between two coverslips, separated by an aluminum washer (thickness 800 μm, inner diameter 12 mm).

Fluorescence fluctuation spectroscopy (FIDA and FCS) experiments were performed on a home‐built confocal microscope described previously [80]. Meanwhile, however, the 561‐nm picosecond pulsed laser was exchanged for a 560‐nm laser (model LDH‐D‐TA‐560B; PicoQuant, Berlin, Germany). Photons were detected by two avalanche photodiodes (COUNT–T100; Laser Components, Berlin, Germany) and registered with their arrival times by using a time‐correlated single photon‐counting (TCSPC) card. After arranging the photon counts in time bins of equal widths, the resulting intensity‐time traces were analyzed to obtain the concentrations and molecular brightness (FIDA) of the observed non‐EV and EV mScarlet‐containing species in the samples and their hydrodynamic radii (FCS). Prior to each measurement, we performed a 300‐s FCS control measurement on a 10‐nm Alexa Fluor 546 dye solution (560‐nm excitation with 2 μW) to measure the focus size.

### FIDA

To acquire data for FIDA from EV samples, we collected image frames consisting of 200 lines containing 200 pixels each by raster‐scanning the confocal observation volume through the CM sample solution. Pixel and line separations were 250 nm; the pixel dwell time was 30 μs. Altogether, 1200 and 2000 frames were recorded in succession on each of the sEV and lEV samples, respectively. The laser power was set to 2 μW for sEVs and 4 μW for lEVs. To determine the molecular brightness of mScarlet (without WNT5a), which is an essential reference parameter for FIDA, we acquired intensity‐time traces on an mScarlet reference sample for 300 s under 2‐μW excitation power with a fixed focus. From these traces, PCHs were calculated by binning the photon counts in 30‐μs intervals, and the molecular brightness of mScarlet was determined by FIDA, using the concentration obtained from an FCS analysis of the same data set as a fixed parameter.

For analysis of raster‐scanned images, the data were filtered against aggregates by using the density‐based spatial clustering of applications with noise (DBSCAN) algorithm [81], which identifies patches of bright pixels due to larger clusters (aggregates) in the images. The pixels recognized by DBSCAN (parameters: search radius, 1.5 and 4 pixels, minimal number of cluster pixels, 6 and 22 for the sEV and lEV samples, respectively) were subsequently discarded from the data pool. Finally, PCHs were compiled, displaying the number of pixels (on a logarithmic scale) as a function of their photon counts registered within the dwell time of 30 μs.

The PCHs were modeled with a theoretical curve based on the FIDA algorithm, using the generating function approach and assuming a 3D Gaussian observation volume [33, 53]. All measured PCHs featured a large, spiky component at low counts, assigned to a single mScarlet molecule. In our FIDA model, it is parameterized by a concentration, *c*_1_, and a molecular brightness, *b*_1_. The broad tails of the PCHs at higher counts arise from the presence of the EVs featuring a wide distribution of the number of associated mScarlet‐WNT5a molecules, *ε*_i_. We adopted a strategy from our previous work on virus‐like particles [53] and heuristically modeled this part of the PCHs by up to four species (*i* = 2–5) with concentrations, *c*_i_, and molecular brightness parameters being multiples of the one of an individual mScarlet, *b*_i_ = *ε*_i_·*b*_1_. A minor contribution of 0.3 kHz due to background (detector shot noise and scattering) was also included. For the determination of the EV parameters, *c*_i_ and *b*_i_, we performed least‐squares fits of the measured PCHs with the FIDA model function. All FIDA model parameters resulting from the fit are compiled in Table S1. In addition, the number of mScarlet‐WNT5A fusion proteins on EVs, *ε*_EV_, and the EV fraction, *f*_EV_, were calculated for each sample as the average of the concentration‐weighted brightness and the sum over the fractions of components 2–5, respectively.

### FCS analysis

The FCS method is based on the measurement of intensity‐time traces of the fluorescence from a tiny volume (~1 fL) in a confocal microscope, from which an autocorrelation curve, $G\left(\tau \right)$, with τ denoting the lag time between all possible pairs of intensities, is computed [54, 82]. Stationarity is a key requirement for the time trace, *i. e*., the mean intensity and its variance must not change with time so that, when measuring multiple traces in succession, the resulting autocorrelation functions are identical within the statistics. Rare events, however, such as the transient appearance of bright aggregates violate stationarity, and it is well known that even a single such event can modify and destroy the autocorrelation curve. The intensity‐time traces of our CM‐derived samples exhibit large spikes due to slowly moving bright particles (Fig. S4A, black segments), which pose serious challenges to FCS analysis.

To cope with this problem, we acquired long traces (1500 s for sEV and 1200 s for lEV samples with laser powers of 2 and 4 μW, respectively, binning time 1 μs) by placing a stationary focus in the sample solution. These traces were broken up into 10‐s segments, and autocorrelation functions were calculated individually for each segment. These are plotted in Fig. S4B, revealing that a sizeable number of autocorrelation traces deviate strongly from the main fraction. To identify and subsequently exclude outliers, we followed an approach similar to the one suggested earlier by Ries *et al*. [83]. After calculating the average, $G_{\mathrm{av}}\left(\tau \right)$, over the individual autocorrelation functions, $G_{j}\left(\tau \right)$, we determined the variance of each trace,

(1) $${\left\langle {\left(G_{j}\left(\tau \right)-G_{\mathrm{av}}\left(\tau \right)\right)}^{2}\right\rangle }_{\tau >1\;\mathrm{ms}},$$

taking only correlation data above 1 ms into account. Outliers, *i.e*., correlation functions with the largest deviations from the mean, were discarded. This procedure was applied iteratively five (four) times for sEV (lEV) samples, leading to a significant reduction of the number of correlation functions (Fig. S4C). Finally, an average $G_{\mathrm{av}}\left(\tau \right)$ was calculated for each sample for further analysis (Fig. 4B).

In FCS, key parameters are extracted by fitting model functions to the experimental correlation functions [82]. Here we chose a sum of *n* components as a model function, representing a fast‐diffusing monomeric species and one (lEV) or two (sEV) more slowly diffusing species.

(2) $$G\left(\tau \right)=\frac{1}{\left\langle N\right\rangle {\left(\sum_{i=1}^{n}f_{i}\;\varepsilon_{i}\right)}^{2}}\sum_{i=1}^{n}f_{i}\;\varepsilon_{i}^{2}\left[1+\frac{F}{\varepsilon_{i}\left(1-F\right)}\exp\left(-\tau /\tau_{\mathrm{pp}}\right)\right]{\left(1+\frac{\tau }{\tau_{D,i}}\right)}^{-1}{\left(1+\frac{1}{S^{2}}\frac{\tau }{\tau_{D,i}}\right)}^{-1/2}.$$

In this equation, $\left\langle N\right\rangle$, $f_{i}$ and $\varepsilon_{i}$ denote the average total number of particles in the observation volume, the fractional weights of the *n* components and their relative molecular brightness values, respectively. The observation volume (~1 fL) is defined by $V_{\mathrm{obs}}=\pi^{3/2}w_{0}^{2}z_{0}$, which assumes a 3D Gaussian shape, with $w_{0}$ and $z_{0}$ being the distances over which the photon collection probability decreases by a factor of $e^{-2}$ (~13.5%) in the lateral and axial directions, respectively. The parameter $w_{0}$ = 290–310 nm was determined on a daily basis by an FCS measurement with a control sample containing the fluorescent dye Alexa 546 with known diffusion coefficient, *D* = 341 μm^2^·s^−1^ [84]. For $\varepsilon_{2}$ and $\varepsilon_{3}$, we took the average values for sEVs and lEVs determined by FIDA as fixed parameters (Table S1); $\varepsilon_{1}=1$. The term in the square brackets accounts for the pronounced flickering of mScarlet between bright and dark states [84] parameterized by the dark fraction, *F*, and the correlation time, $\tau_{\mathrm{pp}}$, of the photophysical dynamics [85]. Notably, because EVs carry several dozen mScarlet markers (as shown by FIDA), their flickering amplitude is strongly suppressed due to averaging. The last two terms in Eq. 1 represent the diffusional correlation decay, with the structure parameter, $S=z_{0}/\omega_{0}$, describing the elongation of the observation volume along the axial direction. This ill‐determined parameter was set to five, as is commonly done [82]. The diffusional correlation time, $\tau_{D,i}$, allows us to calculate the diffusion coefficients, $D_{i}$, and, together with the Stokes‐Einstein relation, the hydrodynamic radii, $R_{H,i}$, of the three diffusing components [54, 82],

(3) $$\tau_{D,i}=\frac{\omega_{0}^{2}}{4D_{i}}=\frac{\omega_{0}^{2}}{4\;}\frac{6\pi \eta R_{H,i}}{k_{B}T},$$

with Boltzmann constant *k*_B_, absolute temperature, *T*, and solution viscosity *η*. We obtained the parameters $f_{i}$ and $\tau_{D,i}$ by fitting the autocorrelation functions with Eq. 2, from which the $R_{H,i}$ parameters of the diffusing entities were calculated with Eq. 3. All fit parameters are given in Table S2.

### Efficiency of chromophore maturation

To determine the fraction of mScarlet proteins carrying a mature functional chromophore, we measured the absorbance of a purified mScarlet solution (expressed in *E. coli*) before and after base denaturation (at pH 13) of a sample. With this well‐established spectroscopic method, which is based on the known extinction coefficients of the mScarlet polypeptide, *ε*_280_ = 34 380 m ^−1^ cm^−1^ and its base‐denatured form carrying a green chromophore, *ε*_447_ = 44 000 m ^−1^·cm^−1^ [86, 87], a maturation efficiency of 0.52 ± 0.01 is obtained from the measured absorbances at 280 and 447 nm.

### Statistical analysis

If not stated otherwise, a two‐tailed Student's *t*‐test was used to examine the statistical significance of differences between data sets.

## Conflict of interest

MB received research grants from Cellzome/GSK and Merck/Darmstadt unrelated to this study.

## Author contributions

AS, CB, GUN, and MB designed the study. AS, AM, OV, MS, MS, NW, MN, and KR performed the experiments. AS, GUN, AK, and KN performed the data analysis. AS, JB, CB, KN, GUN, DJ, LT and MB supervised the study. AS, DK, KN, GUN and MB wrote the manuscript. All authors approved the final version of the manuscript.

## Supporting information


**Fig. S1.** Expression and secretion of WNT5a isoforms in HEK293T cells.
**Fig. S2.** WNT5a Long (WNT5aL) is exported onto two types of EVs.
**Fig. S3.** Fluorescence emission spectra and dynamic light scattering (DLS) data (intensity‐weighted size distributions) of mScarlet‐WNT5a Long‐carrying EVs isolated from conditioned medium (CM) of HEK293T cells.
**Fig. S4.** Fluorescence correlation spectroscopy‐based filtering of autocorrelation curves.
**Fig. S5.** Supplemental western blot data Fig. 1B.
**Fig. S6.** Supplemental western blot data Fig. 3B.
**Fig. S7.** Supplemental western blot data Fig. 3D.
**Fig. S8.** Western blot full scans Fig. S1.
**Fig. S9.** Supplemental western blot data Fig. S2.
**Table S1.** Parameters obtained by fluorescence intensity distribution analysis (FIDA) modeling of the photon counting histograms (PCHs) measured on EV samples.
**Table S2.** Parameters from fitting fluorescence correlation spectroscopy (FCS) autocorrelation curves of the EV samples with Eq. 2.
**Table S3.** Oligonucleotides.
**Table S4.** Plasmids.
**Table S5.** Treatments.
**Table S6.** Antibodies.

## Data Availability

The data that support the findings of this study are available from the corresponding authors [m.boutros@dkfz.de, uli@uiuc.edu] upon reasonable request.
