# Supplementary material for: WNT5a export onto extracellular vesicles studied at single‐molecule and single‐vesicle resolution
Source: FEBS J. 2025 Apr 1;292(17):4631–49. doi: 10.1111/febs.70074 (PMC12414866; doi:10.1111/febs.70074)
Supplement: Supplementary file 1 — Fig. S1. Expression and secretion of WNT5a isoforms in HEK293T cells. Fig. S2. WNT5a Long (WNT5aL) is exported onto two types of EVs. Fig. S3. Fluorescence emission spectra and dynamic light scattering (DLS) data (intensity‐weighted size distributions) of mScarlet‐WNT5a Long‐carrying EVs isolated from conditioned medium (CM) of HEK293T cells. Fig. S4. Fluorescence correlation spectroscopy‐based filtering of autocorrelation curves. Fig. S5. Supplemental western blot data Fig. 1B. Fig. S6. Supplemental western blot data Fig. 3B. Fig. S7. Supplemental western blot data Fig. 3D. Fig. S8. Western blot full scans Fig. S1. Fig. S9. Supplemental western blot data Fig. S2. Table S1. Parameters obtained by fluorescence intensity distribution analysis (FIDA) modeling of the photon counting histograms (PCHs) measured on EV samples. Table S2. Parameters from fitting fluorescence correlation spectroscopy (FCS) autocorrelation curves of the EV samples with Eq. 2. Table S3. Oligonucleotides. Table S4. Plasmids. Table S5. Treatments. Table S6. Antibodies. [file FEBS-292-4631-s001.pdf]

Supporting Information

WNT5a export onto extracellular vesicles studied at single-molecule and single-vesicle resolution

Antonia Schubert[a,b,c,d,e], Ajaree Mongkolsittisilp[f], Andrei Kobitski[f], Matthias Schulz[d], Oksana Voloshanenko[a,b,c], Meike Schaffrinski[d], Nadine Winkler[a,b,c], Michelle Neßling[g], Karsten Richter[g], Dominique Kranz[a], Karin Nienhaus[f], Dirk Jäger[e], Lorenz Trümper[d], Judith Büntzel[d], Claudia Binder[d], Gerd Ulrich Nienhaus[f,h,i,j,*], Michael Boutros[a,b,c,*]

[a] Division Signaling and Functional Genomics, German Cancer Research Center (DKFZ), 69120 Heidelberg, Germany
[b] Department of Cell and Molecular Biology, Medical Faculty Mannheim, Heidelberg University, 69120 Heidelberg, Germany
[c] Institute for Human Genetics, Medical Faculty Heidelberg, Heidelberg University, 69120 Heidelberg, Germany
[d] Department of Hematology and Medical Oncology, University Medical Center Göttingen, 37075 Göttingen, Germany
[e] Department of Medical Oncology, National Center for Tumor Diseases (NCT), University Hospital Heidelberg, 69120 Heidelberg, Germany
[f] Institute of Applied Physics, Karlsruhe Institute of Technology, 76049 Karlsruhe, Germany
[g] Central Unit Electron Microscopy, German Cancer Research Center (DKFZ), 69120 Heidelberg, Germany
[h] Institute of Nanotechnology, Karlsruhe Institute of Technology, 76021 Karlsruhe, Germany
[i] Institute of Biological and Chemical Systems, Karlsruhe Institute of Technology, 76021 Karlsruhe, Germany
[j] Department of Physics, University of Illinois at Urbana-Champaign, Urbana, IL 61801, United States of America

* Corresponding authors:

Michael Boutros, Im Neuenheimer Feld 580, 69120 Heidelberg, Germany, Tel. +49 6221 42 1950, Fax +49 6221 42 1959, E-mail: m.boutros@dkfz.de

Gerd Ulrich Nienhaus, Wolfgang-Gaede-Straße 1, 76131 Karlsruhe, Germany, Tel. +49 0721 608 43401, Fax +49 721 608 48480, E-mail: uli@uiuc.edu

Supplemental Information

Supplemental Figures

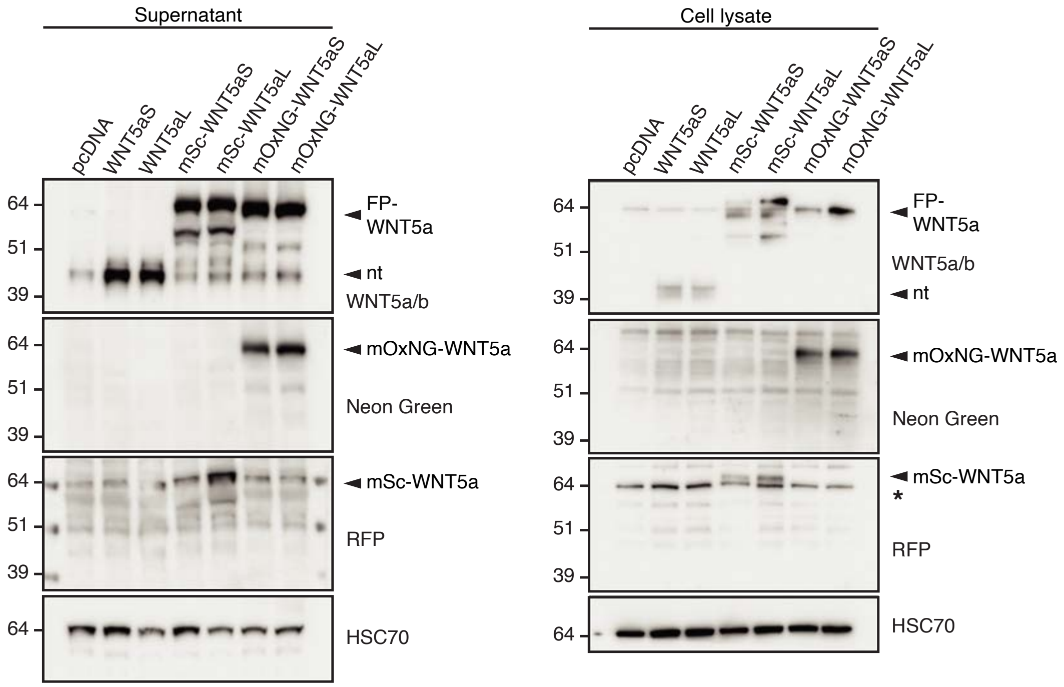

Fig. S1: Expression and secretion of WNT5a isoforms in HEK293T cells. All constructs were expressed in HEK293T cells. Cell lysates were used for immunoblots with the indicated antibodies. Blue Sepharose pulldown and Western blot analysis demonstrate the secretion of the WNT5a constructs Long (WNT6aL) and Short (WNT5aS) into the supernatant. HSC70 serves as a loading control. One of 3 independent experiments is shown. Arrowheads indicate the fluorescently tagged and non-tagged (nt) WNT5a protein. Asterisk marks a non-specific band. mSc = mScarlet, mOxNG = mOxNeonGreen, FP = fluorescent protein, nt = non-tagged. One of more than three independent experiments is shown.

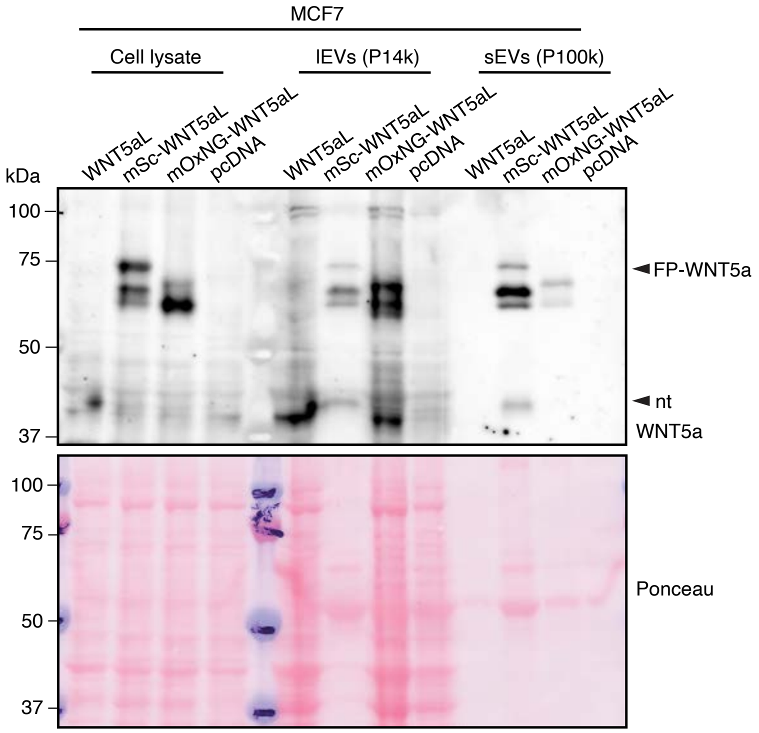

Fig. S2: WNT5a Long (WNT5aL) is exported onto two types of EVs. Western blot analysis of the cell lysate and the pellets P14k (large EVs, lEVs) and P100k (small EVs, sEVs) confirms the export of the WNT5aL constructs onto both EV fractions. Blot for WNT5aL, for WNT5a Short see Fig. 3. Arrowheads indicate the fluorescently tagged and non-tagged (nt) WNT5a protein. Please note that irregularities in protein loading have resulted in missing WNT5aL in the sEV fraction. FP = fluorescent protein, mSc = mScarlet, mOxNG = mOxNeonGreen. Due to high EV yields needed to perform Western blot analysis, Western blots of FP-WNT5a-carrying EVs (Fig. 3D and Fig. S2) were performed once for all FP-WNT5aS and twice for all FP-WNT5aL constructs. Their presence on EVs was confirmed in subsequent experiments employing complementary methodologies, *e.g.*, fluorescence spectroscopy and FCS.

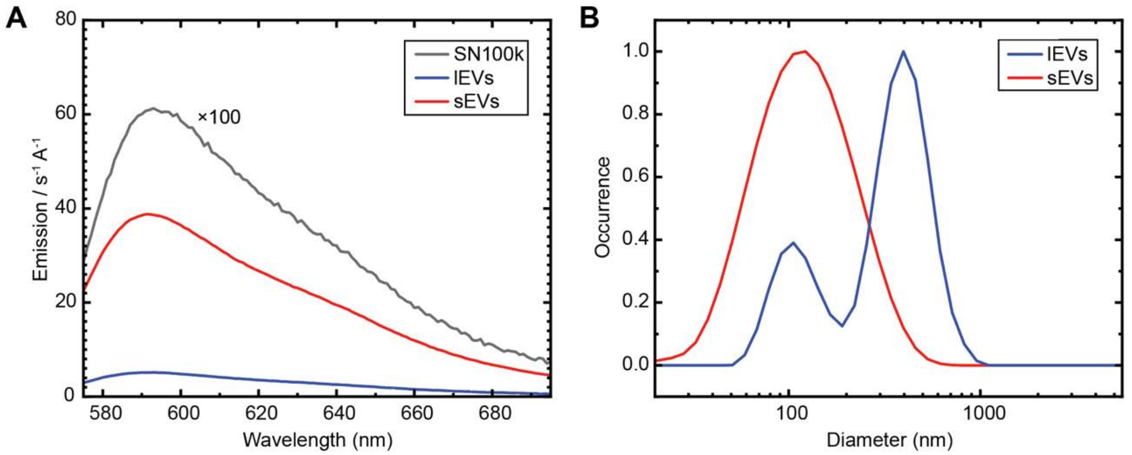

Fig. S3: Fluorescence emission spectra and dynamic light scattering (DLS) data (intensity-weighted size distributions) of mScarlet-WNT5a Long-carrying EVs isolated from conditioned medium (CM) of HEK293T cells. *(A)* Exemplary emission spectra of small EVs (sEVs, red) and large EVs (lEVs, blue) upon excitation at 560 nm. The spectrum of the SN100k fraction (supernatant after centrifugation at 100,000 x g, grey, scaled by a factor of 100) is included for comparison. The areas under the spectra represent the relative amounts of Wnt5a Long if dilution or concentration steps (with respect to CM) are properly accounted for. Notably, while the SN100k fraction was measured directly, the EV fractions were ~1000-fold concentrated by centrifugation and resuspension of the pellet. Therefore, their spectral amplitudes are roughly tenfold enlarged with respect to the 100-fold scaled SN100k spectrum. *(B)* Exemplary intensity-weighted size distributions of sEVs (red) and lEVs (blue). Spectra of one out of more than three independent samples are shown.

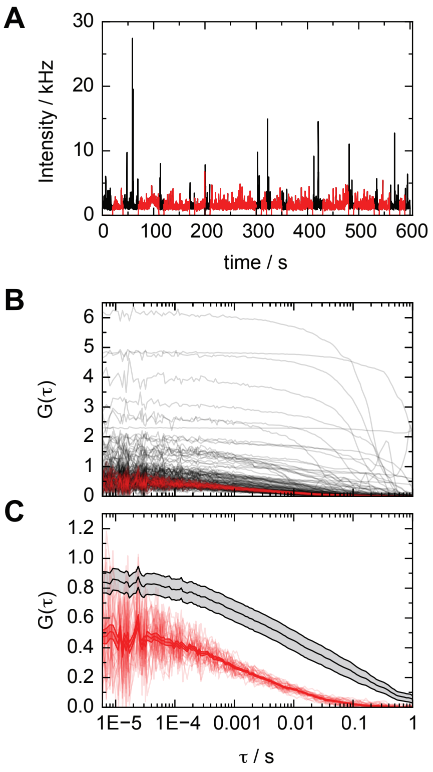

Fig. S4: Fluorescence correlation spectroscopy-based filtering of autocorrelation curves. (*A*) Exemplary intensity-time trace (black) of small EV (sEV) sample A, binned in time intervals of 100 ms for visualization. Only segments colored in red were taken for autocorrelation analysis, those in black were excluded based on their autocorrelation functions markedly deviating from the mean due to intensity spikes in the intensity-time traces. (*B*) Autocorrelation curves calculated for all 10-s segments (color-coded as in panel *A*). (*C*) Thick lines, average autocorrelation curves including (black) all segments and (red) only 67 of 150 curves accepted by the filtering procedure. Comparison of the average correlation shows that long-time correlations are lost upon filtering (as anticipated from panel B). Thin lines, individual correlation functions. Shaded regions around the averages indicate SEM ranges.

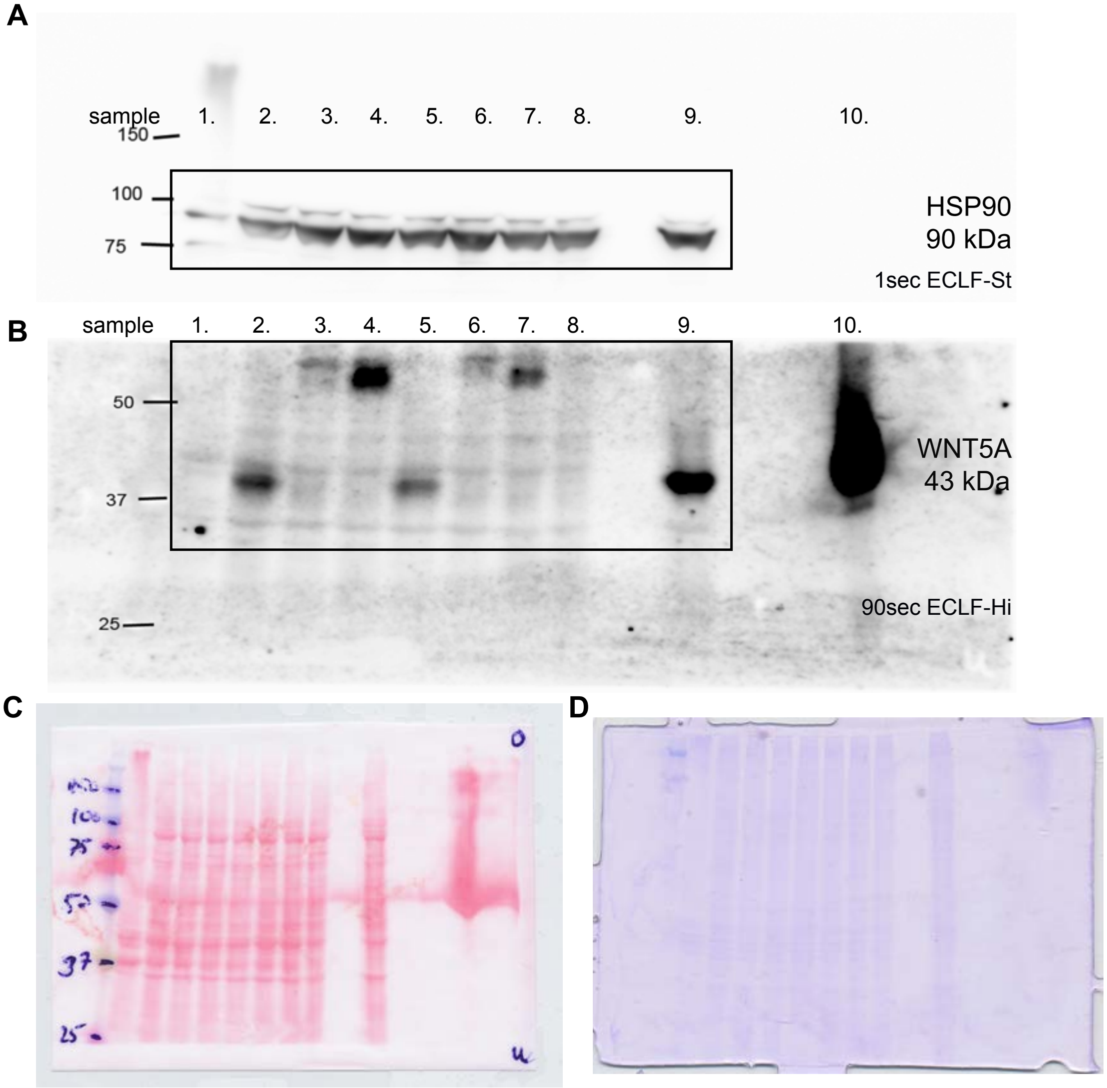

Fig. S5: Supplemental Western blot data Fig. 1B. *(A+B)* Western blot full scans for HSP90 (loading control) and WNT5a, sec refers to exposure time in seconds; ECLF-St/Hi refers to detection reagent used. kDa = kilodalton. Lane descriptions: 1. MCF7 wt (negative loading control), 2. MCF7 WNT5aShort, 3. MCF7 mScarlet-WNT5aShort, 4. MCF7 mOxNeonGreen-WNT5aShort, 5. MCF7 WNT5aLong, 6. MCF7 mScarlet-WNT5aLong, 7. MCF7 mOxNeonGreen-WNT5aLong, 8. MCF7 pcDNA, 9. MDA-MB231 (WNT5a positive loading control), 10. rec. WNT5a (10 ng, positive loading control). Boxes indicate areas shown in Fig. 1B. *(C)* Ponceau staining. *(D)* Coomassie blue staining.

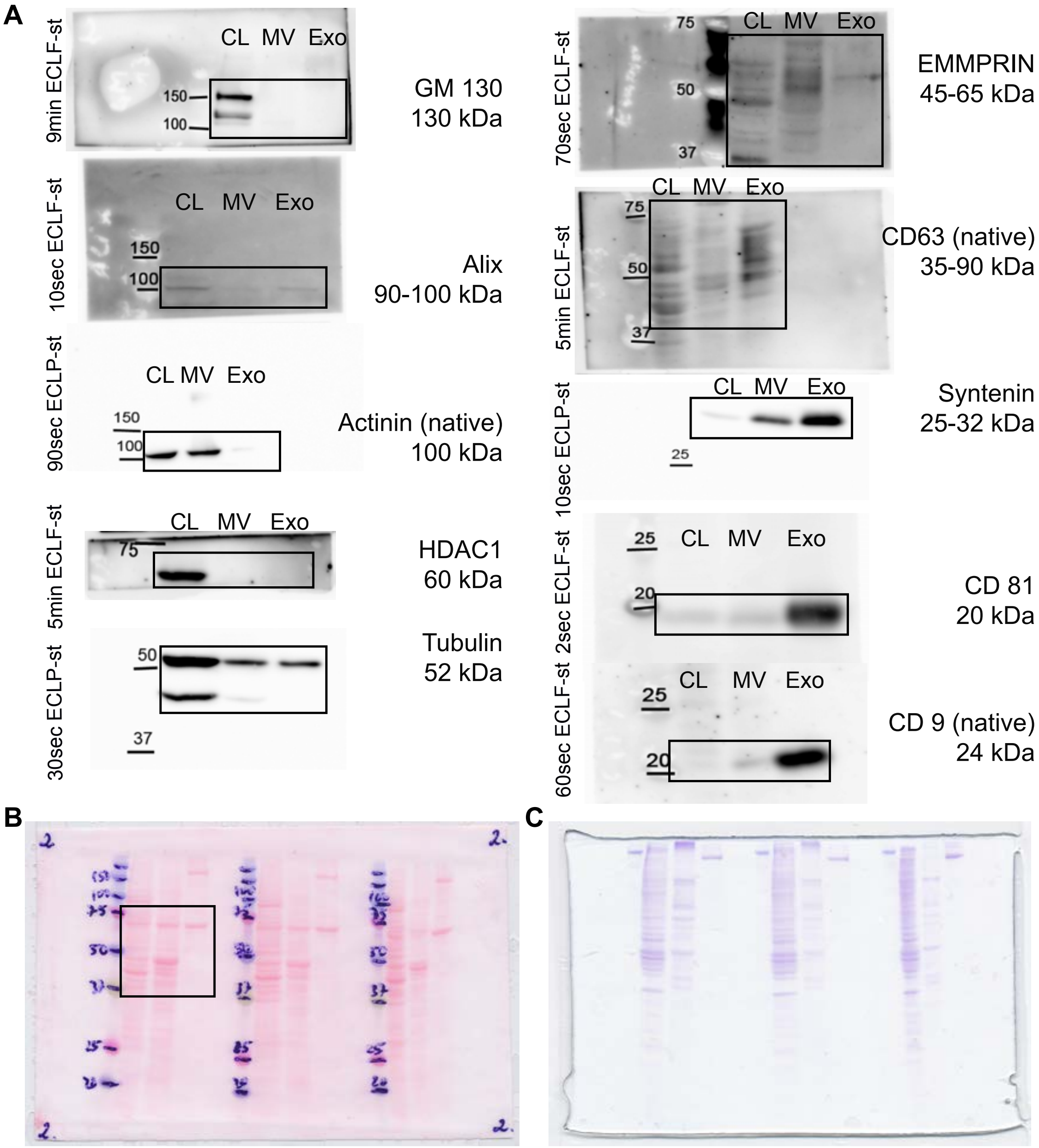

Fig. S6: Supplemental Western blot data Fig. 3B. *(A)* Western blot full scans, CL = cell lysate, MV = large extracellular vesicles (EVs), Exo = small EVs, sec refers to exposure time in seconds; ECLF-st refers to detection reagent used. kDa = kilodalton. *(B)* Ponceau staining. Boxes indicate areas shown in Fig. 3B. *(C)* Coomassie blue staining.

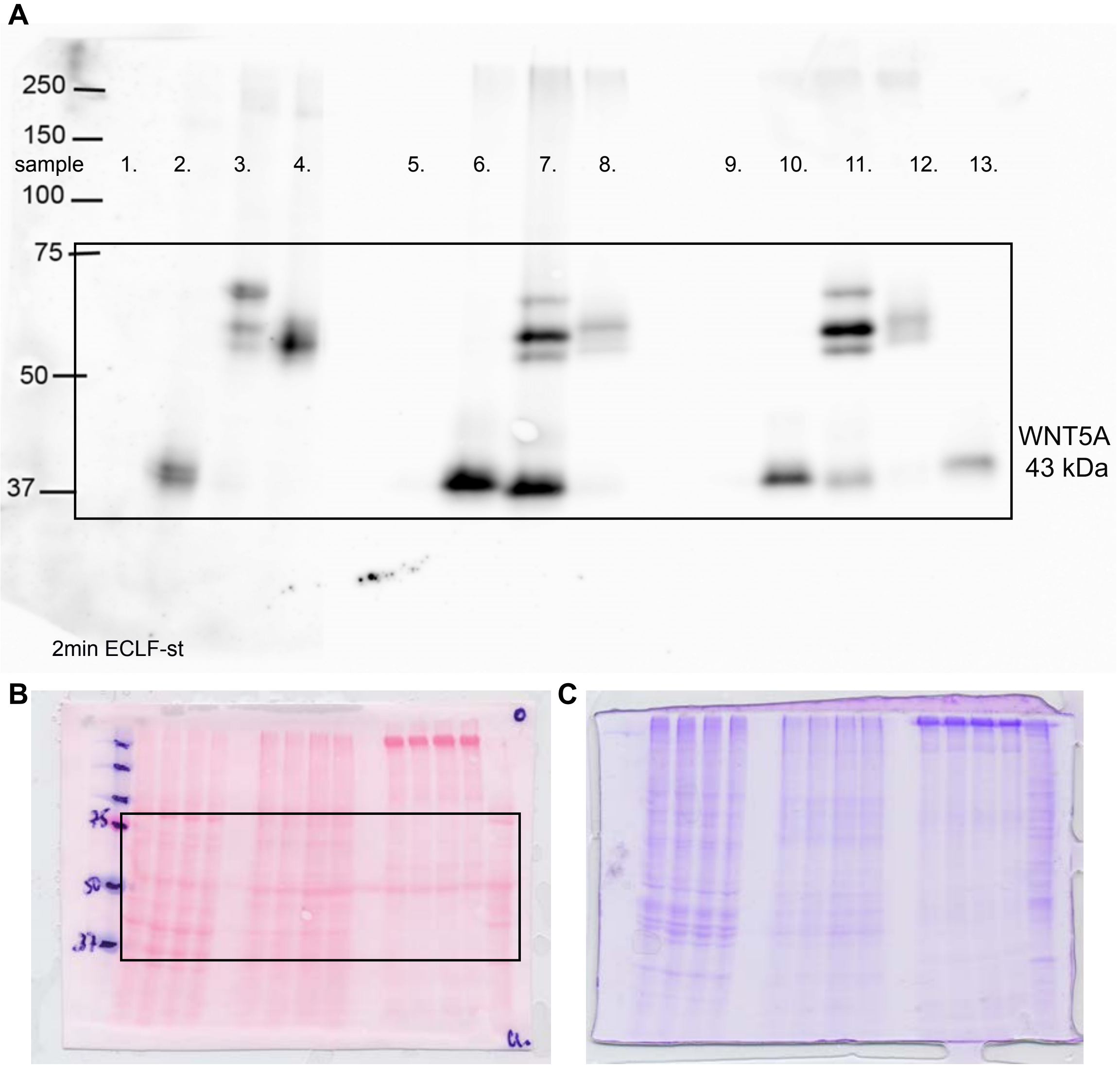

Fig. S7: Supplemental Western blot data Fig. 3D. *(A)* Western blot full scan, min refers to exposure time in minutes; ECLF-st refers to detection reagent used. kDa = kilodalton. Lane descriptions: 1. Cell lysate MCF7 pcDNA, 2. Cell lysate MCF7 WNT5aShort, 3. Cell lysate MCF7 mScarlet-WNT5aShort, 4. Cell lysate MCF7 mOxNeonGreen-WNT5aShort, 5. Large EVs (lEVs) MCF7 pcDNA, 6. lEVs MCF7 WNT5aShort, 7. lEVs MCF7 mScarlet-WNT5aShort, 8. lEVs MCF7 mOxNeonGreen-WNT5aShort, 9. Small EVs (sEVs) MCF7 pcDNA, 10. sEVs MCF7 WNT5aShort, 11. sEVs MCF7 mScarlet-WNT5aShort, 12. sEVs MCF7 mOxNeonGreen-WNT5aShort, 13. Cell lysate MDA-MB231 (WNT5a positive loading control). *(B)* Ponceau staining. Boxes indicate areas shown in Fig. 3D. *(C)* Coomassie blue staining.

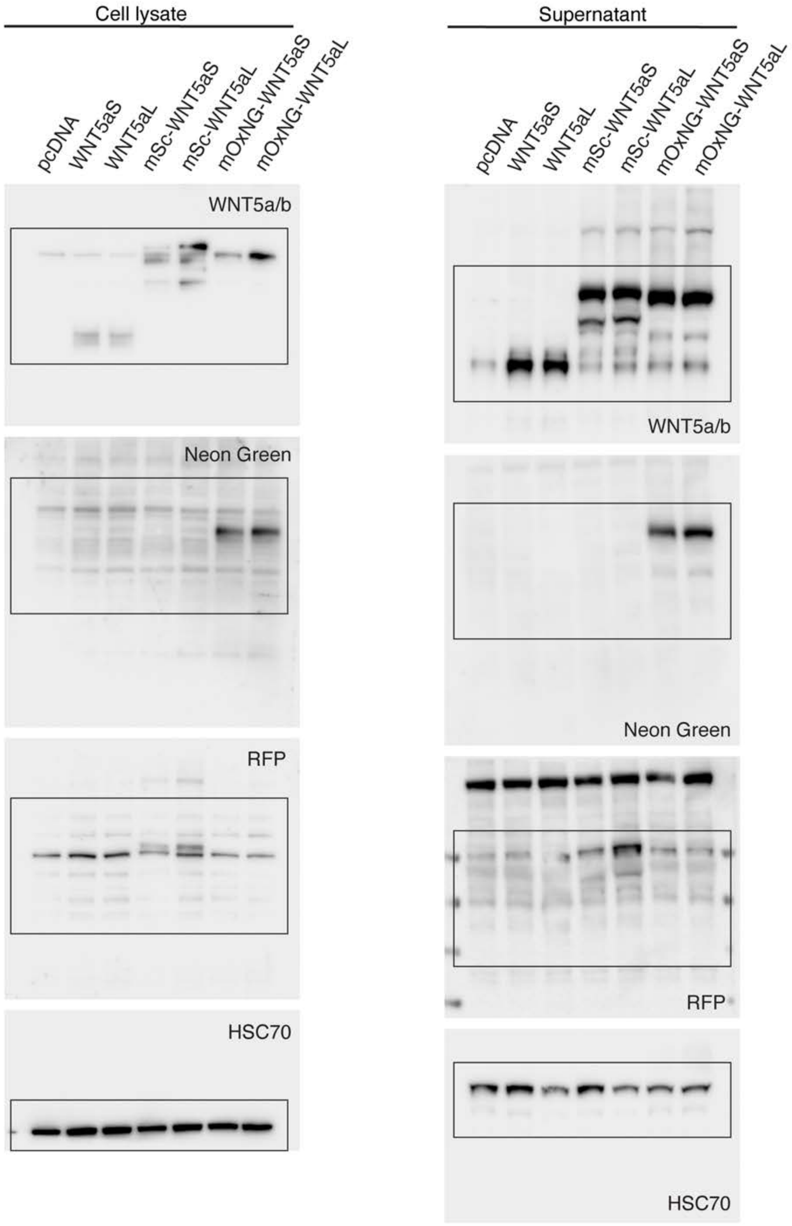

Fig. S8: Western Blot Full Scans Fig. S1. Boxes indicate areas shown in Fig. S1.

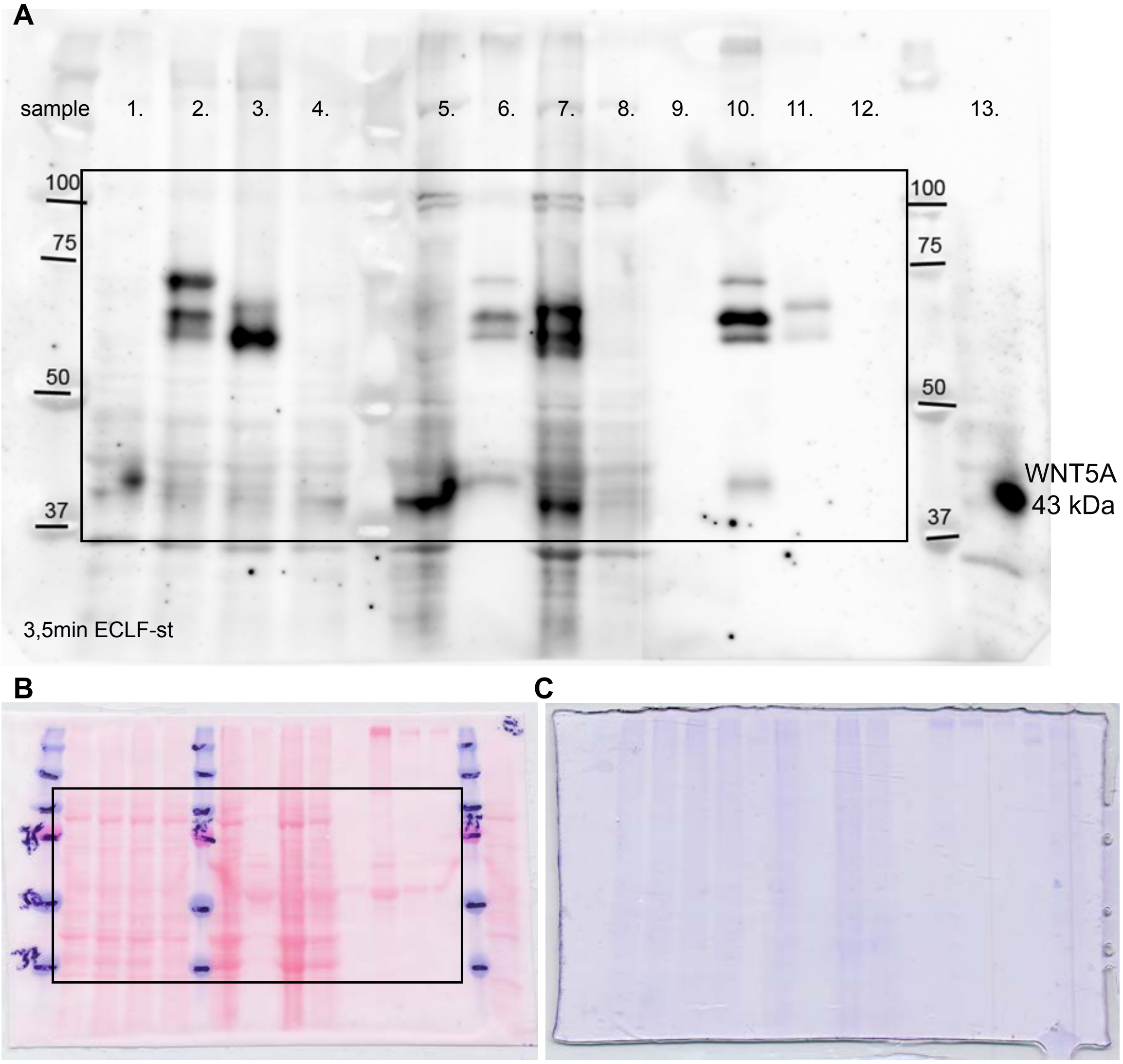

Fig. S9: Supplemental Western blot data Fig. S2. *(A)* Western blot full scan, min refers to exposure time in minutes; ECLF-st refers to detection reagent used. kDa = kilodalton. Lane descriptions: 1. Cell lysate MCF7 WNT5aLong, 2. Cell lysate MCF7 mScarlet-WNT5aLong, 3. Cell lysate MCF7 mOxNeonGreen-WNT5aLong, 4. Cell lysate MCF7 pcDNA, 5. Large EVs (lEVs) MCF7 WNT5aLong, 6. lEVs MCF7 mScarlet-WNT5aLong, 7. lEVs MCF7 mOxNeonGreen-WNT5aLong, 8. Large EVs MCF7 pcDNA, 9. Small EVs (sEVs) MCF7 WNT5aLong, 10. sEVs MCF7 mScarlet-WNT5aLong, 11. sEVs MCF7 mOxNeonGreen-WNT5aLong, 12. sEVs MCF7 pcDNA, 13. Cell lysate MDA-MB231 (WNT5a positive loading control). *(B)* Ponceau staining. Boxes indicate areas shown in Fig. S2. *(C)* Coomassie blue staining.

Table S1: Parameters obtained by fluorescence intensity distribution analysis (FIDA) modeling of the photon counting histograms (PCHs) measured on EV samples.

			**large EV samples**				**small EV samples**		
			**Sample 1**	**Sample 2**	**Sample 3**		**Sample 1**	**Sample 2**	**Sample 3**
Brightness (species 1[a])	b_1 / kHz	F[b]	2.0[c]	2.0[c]	2.0[c]	F	1.5[d]	1.5[d]	1.5[d]
Concentration (species 1)	c_1 / nM		4 ± 1	5 ± 1	12 ± 3		0.5 ± 1.4	3.0 ± 2.4	2.2 ± 1.6
Brightness (species 2[a])	b_2 / kHz	F	100	100	100	F	75	75	75
Concentration (species 2)	c_2 / pM		9 ± 3	37 ± 8	87 ± 18		6 ± 5	16 ± 10	13 ± 5
Brightness (species 3[a])	b_3 / kHz	F	600	600	600	F	270	270	270
Concentration (species 3)	c_3 / pM		0.21 ± 0.06	0.8 ± 0.2	2.7 ± 0.2		0.3 ± 0.2	0.6 ± 0.2	0.20 ± 0.06
Brightness (species 4[a])	b_4 / kHz	F	1200	1200	1200	F	900	900	900
Concentration (species 4)	c_4 / pM		0.01 ± 0.04	0.12 ± 0.02	0.36 ± 0.07		0.017 ± 0.005	0.007 ± 0.002	0.030 ± 0.001
Brightness (species 5[a])	b_5 / kHz	F	2400	2400	2400		N/A	N/A	N/A
Concentration (species 5)	c_5 / pM		0.07 ± 0.01	0.29 ± 0.02	0.29 ± 0.02		N/A	N/A	N/A
Fractional amount of EVs[e]	$f_{EV} \times 10^3$		2.5 ± 1.0	7.5 ± 2.4	7.4 ± 2.3		11.1 ± 30.5	5.5 ± 5.3	6.0 ± 4.9
Mean EV brightness[f]	ε_{EV}		63.4 ± 4.3	65.9 ± 3.2	63.4 ± 2.8		58.6 ± 8.3	54.9 ± 3.2	53.1 ± 1.4
SD[g] of EV brightness			107.2 ±	109.8 ±	84.5 ± 8.5		39.5 ± 16.0	26.7 ± 8.5	30.5 ± 5.8
Average EV brightness	$\langle \varepsilon_{EV} \rangle$		64 ± 2				56 ± 3		
Average SD[g]			100.5 ± 8.0				32.2 ± 6.3		

[a] Species 1, monomeric mScarlet; species 2 – 5, EV particles.

[b] F, fixed parameter.

[c] Monomer brightness, calculated from the monomer brightness for small EV samples by adjusting for different laser powers (factor 2) and the transmission ratio of the different bandpass filters ($T_{600/37}$ / $T_{609/62}$ = 0.67) used in the lEV and sEV measurements.

[d] Monomer brightness was determined by FIDA analysis of a sample of mScarlet, using the concentration obtained by FCS analysis on the same sample.

[e] Fractional amount of EV particles (species 2 – 5).

[f] Overall EV brightness (mean ± SEM) relative to mScarlet (*i.e.*, number of fluorescing mScarlet-WNT5a molecules per EV),
calculated by concentration-weighted averaging over components 2 – 5.

[g] SD, standard deviation.

Data were taken on three independent small EV and three independent large EV samples (samples 1 – 3).

Table S2: Parameters from fitting fluorescence correlation spectroscopy (FCS) autocorrelation curves of the EV samples with Eq. 2.

			large EV samples				small EV samples		
			**Sample A**	**Sample B**	**Sample C**		**Sample A**	**Sample B**	**Sample C**
**mScarlet (monomer)**									
Number of molecules in observation volume	N		1.90 ± 0.01	2.06 ± 0.02	2.20 ± 0.02		6.9 ± 1.8	4.4 ± 0.4	4.6 ± 1.2
Correlation time of the photophysical dynamics	τ_{pp} / µs	F[a]	200[b]	200[b]	200[b]	F	200[c]	200[c]	200[c]
Dark fraction	F	F	0.5[b]	0.5[b]	0.5[b]	F	0.36[c]	0.36[c]	0.36[c]
**EV component 1**									
Fractional weight	f_1[d]		1	0.999957	0.999922		0.99793	0.99895	0.9987
Diff. correlation time	$\tau_{D,1}$ / µs	G[e]	428 ± 17	428 ± 17	428 ± 17	F	428[f]	428[f]	428[f]
Rel. molecular brightness	ε_1	F	1	1	1	F	1	1	1
**EV component 2**									
Fractional weight	f_2	F	0	0	0		(13.4 ± 6.0) × 10^{-4}	(7.9 ± 1.4) × 10^{-4}	(7 ± 4) × 10^{-4}
Diff. correlation time	$\tau_{D,2}$ / ms		N/A	N/A	N/A		0.9 ± 0.2	1.7 ± 0.1	0.7 ± 0.3
Rel. molecular brightness	ε_2		N/A	N/A	N/A	F	56[g]	56[g]	56[g]
**EV component 3**									
Fractional weight	f_3		(1 ± 2) × 10^{-6}	(43 ± 3) × 10^{-6}	(78 ± 4) × 10^{-6}		(7.3 ± 3.0) × 10^{-4}	(2.6 ± 0.5) × 10^{-4}	(6 ± 2) × 10^{-4}
Diff. correlation time	$\tau_{D,3}$ / ms	G	10.4 ± 1.5	10.4 ± 1.5	10.4 ± 1.5	G	16.2 ± 1.4	16.2 ± 1.4	16.2 ± 1.4
Rel. molecular brightness	ε_3	F	64[g]	64[g]	64[g]	F	64[g]	64[g]	64[g]
Diff. correlation time	τ_{av} / ms[h]		N/A	N/A	N/A		5.6 ± 2.3	4.0 ± 0.8	9.8 ± 2.5

[a] F, fixed parameter.
[b] Parameters obtained from fits of autocorrelation curves of mScarlet in buffer (4 µW excitation).
[c] Parameters obtained from fits of autocorrelation curves of mScarlet in buffer (2 µW excitation).
[d] $f_1 = 1 - f_2 - f_3$
[e] G, global parameter (shared in the simultaneous fit of the three small EV and large EV curves).
[f] Parameter taken from a global fit of the correlation curves of the large EV samples.
[g] Determined by FIDA.
[h] Weighted average of $\tau_{D,1}$ and $\tau_{D,2}$.

Data were taken on three independent small EV and three independent large EV samples (samples A – C).

Table S3: Oligonucleotides

Cloning			
**Construct**	**Name**	**Sequence 5' to 3'**	**Description**
pcDNA mScarlet/mOxNeonGreen-WNT5a LONG	pcDNA WNT5a mScarlet fwd	aaaaagcaggctccgcggccATGAAGAAGTCCATTGGAATATTAAGCCCAGGAGTT	InFusion cloning pcDNA -mScarlet-WNT5a LONG and pcDNA mOxNeonGreen-WNT5a LONG, Insert 1 (Signal peptide), forward primer
pcDNA mScarlet-WNT5a LONG	HA1NheImScarlet rev	atcactgcctcgcccttgctcaccatGCTAGCTTCAATTACAACCTGGGCGAAGGAG	InFusion cloning pcDNA mScarlet-WNT5a LONG, Insert 1 (Signal peptide), reverse primer
	HA1NheImScarlet fwd	TTCTCCTTCGCCCAGGTTGTAATTGAAGCTAGCatggtgagcaagggcgaggcagtgatcaa	InFusion cloning pcDNA mScarlet-WNT5a LONG, Insert 2 (mScarlet), forward primer
	pcDNA WNT5a mScarlet Linker BspEI Exon2 Exon3 rev	ctgaacagggttattcatacctagcgaccaccaaGAATTGGCTCCGGAGCCGGAGCCTCCcttgtacagctcgtccatgccgccggt	InFusion cloning pcDNA mScarlet-WNT5a LONG, Insert 2 (mScarlet), reverse primer
pcDNA mOxNeonGreen-WNT5a LONG	HA1NheImOXNeonGreen rev	TTGTCTTCTTCTCCCTTTGAGGACATGCTAGCTTCAATTACAACCTGGGCGAAGGAG	InFusion cloning pcDNA mOxNeonGreen-WNT5a LONG, Insert 1 (Signal peptide), reverse primer
	HA1NheImOXNeonGreen fwd	TTCGCCCAGGTTGTAATTGAAGCTAGCATGTCCTCAAAGGGAGAAGAAGACAACA	InFusion cloning pcDNA mOxNeonGreen-WNT5a LONG, Insert 2 (mScarlet), forward primer
	Exon2BspEILinkermOXNeon rev	gaccaccaaGAATTGGCTCCGGAGCCGGAGCCTCCCTTATACAGTTCGTCCATCCCCAT	InFusion cloning pcDNA mOxNeonGreen-WNT5a LONG, Insert 2 (mScarlet), reverse primer

pcDNA WNT5a SHORT	pcDNA WNT5a SHORT Fragment 1.FOR	aaaaagcaggctccgcggccatggctggaagtgcaatgtctTCC	InFusion cloning pcDNA WNT5a SHORT Insert 1 (shortened Signal peptide), forward primer
	pcDNA WNT5a SHORT Fragment 3.REV	TCACCGCGTAtgtgaaggccgtctcgcggctgcctatctgcaTCACCCTGCCAAAAACAG	InFusion cloning pcDNA WNT5a SHORT Insert 1 (shortened Signal peptide), reverse primer
pcDNA mScarlet-WNT5a SHORT	pcDNA WNT5a SHORT mScarlet Fragment 1.REV	CTGCCTCGCCCTTGCTCACCATGCTAGCatatacttctgacatctgaacagggttattcatacCT	InFusion cloning pcDNA mScarlet-WNT5a SHORT Insert 1 (Signal peptide), reverse primer
	pcDNA WNT5a Fragment2 mScarlet.FOR	ccctgttcagatgtcagaagtatatGCTAGCatggtgagcaagggcgagg	InFusion cloning pcDNA mScarlet-WNT5a SHORT Insert 2 (mScarlet), forward primer
	pcDNA WNT5a SHORT mScarlet Fragment2.REV	tgcagagaggctgtgctcctataatTCCGGAGCCGGAGCCTCCcttgtacagctcgtccatgccg	InFusion cloning pcDNA mScarlet-WNT5a SHORT Insert 2 (mScarlet), reverse primer
	pcDNA WNT5a SHORT mScarlet Fragment 3.FOR	GCATGGACGAGCTGTACAAGGGAGGCTCCGGCTCCGGAattataggagcacagcctctctgCA	InFusion cloning pcDNA mScarlet-WNT5a SHORT Insert 3, forward primer
pcDNA mOxNeonGreen-WNT5a SHORT	pcDNA WNT5a SHORT mOxNeonGreen Fragment 1.REV	TCTTCTTCTCCCTTTGAGGACATGCTAGCatatacttctgacatctgaacagggttattcatacCT	InFusion cloning pcDNA mOxNeonGreen-WNT5a SHORT, Insert 1 (Signal peptide), reverse primer
	pcDNA WNT5a SHORT mOxNeonGreen Fragment 2.FOR	tgttcagatgtcagaagtatatGCTAGCATGTCCTCAAAGGGAGAAGAAGACAACA	InFusion cloning pcDNA mOxNeonGreen-WNT5a SHORT, Insert 2 (mOxNeonGreen), forward primer

	pcDNA WNT5a SHORT mOxNeonGreen Fragment 2.REV	cagagaggctgtgctcctataatTCCGGAGCCGGAGCCTCCCTTATACAGTTCGTCCATCCCCATCACAT	InFusion cloning pcDNA mOxNeonGreen-WNT5a SHORT, Insert 2 (mSOxNeonGreen), reverse primer
	pcDNA WNT5a SHORT mOxNeonGreen Fragment 3.FOR	GGATGGACGAACTGTATAAGGGAGGCTCCGGCTCCGGAattataggagcacagcctctctgC	InFusion cloning pcDNA mOxNeonGreen-WNT5a SHORT, Insert 3, forward primer

Sequencing

Name	Sequence 5' to 3'	Description
mScarlet_M_rev	Cttggtgaaggccctgga	Sequencing primer, mScarlet, reverse

Table S4: Plasmids

Name	Alternative Name	Source	Reference
pcDNA	pcDNA3.2/V5-DEST	Invitrogen	
renilla luciferase	pAct-RL, Renilla luciferase	D. Nickles, M.B. laboratory	[85]
TCF4/WNT firefly luciferase	6xKD; pGL4.26 6xTcf-Firefly luciferase	K. Demir, M.B. laboratory	[86]
WNT3a	pcDNA WNT3a	Addgene #35908	[87]
WNT5a (LONG)	pcDNA WNT5a	Addgene #35911	[87]
mSc(arlet)-WNT5a L(ONG)	pcDNA WNT5a LONG-mScarlet	-	this publication
mOxNG (NeonGreen)-WNT5a L(ONG)	pcDNA WNT5a LONG-mOxNeonGreen	-	this publication
WNT5a SHORT	pcDNA WNT5a SHORT	-	this publication
mSc(arlet)-WNT5a SHORT	pcDNA WNT5a SHORT-mScarlet	-	this publication
mOxNG (NeonGreen)-WNT5a SHORT	pcDNA WNT5a SHORT-mOxNeonGreen	-	this publication

Table S5: Treatments

Name	Description	Supplier
WNT3a	Recombinant mouse WNT3A protein	PeproTech 315-20
WNT5a	Recombinant Human/Mouse WNT5a protein (gene ID 7474 (human), no information on the isoform)	645-WN-010/CF, R&D System

Table S6: Antibodies

Target	Species	Supplier	Catalogue Number	Usage	Dilution
Primary antibodies					
β-actin - HRP	mouse	Santa Cruz Biotechnology	sc47778-HRP	Western Blot	1:1000 -1:2000
HSC70	mouse	Santa Cruz	7298	Western Blot	1:1000
HSC90	mouse	SantaCruz	sc-13119,	Western Blot	1:1000
mCherry	mouse	Abcam	ab125096	Western Blot	1:1000
mNeonGreen	mouse	Chromotek	[32F6]	Western Blot	1:1000
RFP	mouse	Chromotek	[6G6]	Western Blot	1:1000
RFP	rabbit	Genetex	GTX127897-100	Western Blot	1:1000
WNT3a	rabbit	GeneTex	GTX128101	Western Blot	1:1000
WNT5a	rat	R&D	MAB645	Western Blot	1:1000
WNT5a/b (C27E8)	rabbit	Cell Signaling Technology	2530	Western Blot	1:1000
Secondary antibodies					
mouse IgG - HRP	goat	Jackson Immuno Research	115-035-003	Western Blot	1:2000
rabbit IgG - HRP	goat	Jackson Immuno Research	111-035-003	Western Blot	1:2000
rat IgG - HRP	goat	Santa Cruz	Sc-2006	Western Blot	1:10000
